# The Role of Senescence, its Therapeutic Relevance and Clinical Implications in the Tumor Microenvironment

**DOI:** 10.7150/thno.112633

**Published:** 2025-07-28

**Authors:** Hanzhe Shi, Mingming Xiao, Yangyi Li, Xiyu Liu, Jintong Na, Chen Liang, Jie Hua, Qingcai Meng, Miaoyan Wei, Wei Wang, Jin Xu, Xianjun Yu, Si Shi

**Affiliations:** 1 Department of Pancreatic Surgery, Fudan University Shanghai Cancer Center, Shanghai, 200032, China.; 2 Department of Oncology, Shanghai Medical College, Fudan University, Shanghai, 200032, China.; 3 Shanghai Pancreatic Cancer Institute, Shanghai, 200032, China.; 4 Shanghai Key Laboratory of Precision Medicine for Pancreatic Cancer, Shanghai, 200032, China.; 5 Pancreatic Cancer Institute, Fudan University, Shanghai, 200032, China.; 6 State Key Laboratory of Targeting Oncology, Guangxi Medical University, Nanning, Guangxi, 530021, China.; 7 National Center for International Research of Bio-targeting Theranostics, Guangxi Medical University, Nanning, Guangxi, 530021, China.

**Keywords:** cellular senescence, aging, tumor microenvironment, senescence-associated secretory phenotype, cancer treatment

## Abstract

Cellular senescence is characterized by cell cycle arrest, resistance to apoptosis, the expression of senescence markers, and the acquisition of senescence-associated secretory phenotype (SASP). In this review, we discuss the role of cellular senescence within the tumor microenvironment. Some senescent innate immune cells fail to sustain their antitumor function and may even promote tumor progression. Senescent CD8^+^ and CD4^+^ T cells become dysfunctional and are implicated in immunosuppression, angiogenesis, and resistance to immunotherapy. Research on stromal senescence primarily focuses on the SASP. The SASP functions as a double-edged sword. It promotes immune surveillance in the early stages of a tumor while inhibiting tumor immunity in its advanced stages. Strategies to target senescence in cancer therapies include four main approaches: inducing senescence, inhibiting tumor-promoting SASP, clearing senescent cells, and reversing senescence. Although not yet in clinical practice, these approaches hold promise for future cancer treatments.

## 1. Introduction

Aging is an inevitable biological process that all humans experience. Given the global impact of aging, advancing research into aging and age-related diseases is crucial. Numerous studies widely acknowledge that cancer is associated with age, demonstrating increased susceptibility among older individuals 1. Indeed, the hallmarks of aging and cancer share remarkable similarities. Kroemer *et al.* have identified meta-hallmarks common to both aging and cancer, including genomic instability, epigenetic alterations, dysbiosis, and chronic inflammation 2. Cellular senescence was first described in the 1960s when human fibroblasts exhibited a decline in proliferative capacity after numerous cell cycles in vitro 3. Arne N Akbar and Sian M Henson have outlined the three phases of senescence induction: induction by stimuli, DNA damage response, and growth arrest 4. In 2022, Douglas Hanahan introduced four new hallmarks to the previously established ten hallmarks of cancers 5, including the presence of senescent cells 6. This underscores the critical importance of research on cellular senescence within the TME.

The tumor microenvironment (TME) is the habitat in which tumor cells live and proliferate. Beyond neoplastic cells, the TME encompasses a heterogeneous assemblage of innate and adaptive immune populations, cancer-associated fibroblasts, endothelial cells, mesenchymal stromal cells, and resident stem-like cells. Numerous reviews have established the link between the senescence of tumor cells and the onset and progression of cancers 41-45. Therefore, our work will mainly focus on the senescence of non-cancerous components within the tumor microenvironment. In particular, accumulating evidence highlights the induction of senescence in both immune cells and stromal compartments 46-50. Senescence exerts multifaceted effects on antitumor immunity. On the one hand, senescent cells secrete chemokines and surface ligands that recruit and activate immune surveillance 7-9; on the other, senescent immune cells may become dysfunctional. Senescence may assist in evading immune clearance through transient cell-cycle re-entry or the release of immunosuppressive factors, thereby fostering resistance to therapy and adverse clinical outcomes. Deconvoluting this paradox is essential for a better understanding of senescence's dualistic roles within TME. Table 1 summarizes the common inductions of cellular senescence within the TME, their triggers, senescent cells involved, and their roles within TME **(Table 1)**. Here, we review the changes in senescence within innate immunity, adaptive immunity, and stroma. We will elaborate on their contributions to tumor progression and cancer therapies, and the extent to which patients may benefit from targeting senescent cells within TME.

## 2. Aging, Immunosenescence, and Cellular Senescence

In 2013, Kroemer *et al.* proposed nine molecular hallmarks of aging, with cellular senescence as one of them 51. This underscores the link between physiological aging and cellular senescence. Cellular senescence denotes a state of permanent proliferative arrest that cells enter following extended in vitro replication or upon exposure to sublethal stressors or oncogenic stimuli 52. Senescent cells exhibit several characteristics: morphological abnormalities, irreversible cell cycle arrest, apoptosis resistance, expression of senescence markers, mitochondrial dysfunction, metabolic alterations, and the acquisition of senescence-associated secretory phenotype (SASP) 52, 53. The onset of senescence is triggered by a variety of insults—irreparable DNA damage, telomere attrition, mitochondrial perturbations, metabolic derangements, and oncogene activation—all of which accrue with chronological aging 54. Consequently, cells subjected either to a finite replicative lifespan or to diverse stressors during organismal aging undergo senescence, which in turn contributes to the pathogenesis of multiple age-related disorders. Specifically, metabolism, mitochondrial function, and senescence are interrelated in a bidirectional manner, each influencing and being influenced by the others. Senescent cells exhibit hallmark metabolic alterations, such as heightened aerobic glycolysis, sustained tricarboxylic acid (TCA) cycle activity, increased glutaminolysis, and lipid accumulation 55, 56. For example, glycogen overload elevates reactive oxygen species (ROS), precipitating senescence 57, whereas methionine deprivation induces DNA damage-mediated senescence 58. On the other hand, mitochondrial dysfunction—manifested as reduced respiratory capacity and membrane potential, aberrant organelle biogenesis, and mtDNA mutations—drives cells into senescence 59.

Senescence resulting from repeated cell divisions is termed replicative senescence (RS) 3, driven by telomere shortening. Stress-induced premature senescence (SIPS) encompasses senescence triggered by stress signals such as oncogene activation, hypoxia, and DNA damage 60-62. Specifically, cellular senescence induced by treatments such as radiation, conventional chemotherapies, or targeted therapies is termed therapy-induced senescence (TIS) 16, 43, 63. Senescence induced by the aberrant activation of oncogenic signaling is termed oncogene-induced senescence (OIS) 64. TIS and OIS are both categorized into SIPS.

Differentiating cellular senescence from immunosenescence is critical, as these interrelated yet distinct phenomena both drive organismal aging and age-related pathology. Cellular senescence denotes a cell-intrinsic, irreversible proliferative arrest, whereas immunosenescence refers to the age-associated, systemic decline of immune competence across both innate and adaptive immunity. Immunosenescence can result from thymic involution, persistent antigen exposure, chronic inflammation, etc. 49, 65-68. Importantly, cellular senescence of immune cells partly contributes to immunosenescence 48, 49. Among these factors, thymic involution represents the most prominent and specific change associated with immunosenescence 69, 70. As individuals age, thymic involution leads to thymic atrophy, reduction in thymocytes, and a decreased output of naïve T cells 69, 70. Subsequently, older individuals may experience an altered phenotype of peripheral T cells, replicative senescence, and ultimately dysfunction in adaptive immunity 71, potentially leading to a higher mortality 72. Concurrently, 'inflammaging'—a state of sterile, chronic, low-grade inflammation driven predominantly by innate immune cells—both contributes to and is exacerbated by immunosenescence 73. Some researchers believe that inflammaging is a component of physiological aging. Once influenced by frail gene variants, it may lead to age-related diseases, termed as 'Second hit theory' 74. Some may view inflammaging as the counterpart to immunosenescence 75, with each promoting the other. Although senescent cells potentiate inflammaging via pro-inflammatory SASP factors, they represent only one facet of this multifactorial process, which also encompasses accrual of cellular debris, accumulation of damage-associated molecular patterns (DAMPs), and a decline in proteasomal and autophagic clearance mechanisms 41, 76. Moreover, immunosenescence will drive systemic aging 77. Researchers have modeled physiological immunosenescence by knocking out Ercc1, a gene encoding a specific DNA repair protein, to reveal the senescence of non-lymphoid organs 77, highlighting the interaction between immunosenescence and aging.

## 3. Tumor Cell Senescence: Friend or Foe?

### 3.1. Senescence and Cancer Prior to Oncogenesis

Aging elevates oncogenic risk through chronic inflammation, genomic instability, dysbiosis, and epigenetic drift 78. Under genotoxic stress—such as DNA damage or aberrant oncogene activation—normal cells either undergo apoptosis or enter a permanent growth arrest termed senescence, thereby acting as a potent tumor-suppressive barrier 79. Mitochondrial pyruvate dehydrogenase (PDH) activation acts as a pivotal metabolic mechanism driving oncogene-induced senescence (OIS), linking enhanced mitochondrial respiration and redox stress to OIS-driven tumor suppression. This indicates the significant role of mitochondrial function in cellular senescence. Moreover, senescent cells propagate senescence to neighboring cells via paracrine SASP factors and juxtacrine signals 44, and are cleared by immune cells, a process called senescence surveillance 44, 45, 80. For example, pre-malignant hepatocytes undergoing OIS are cleared by macrophages recruited through CD4⁺ T-cell-derived SASP chemokines, and both CD4⁺ and CD8⁺ T cells can mediate senescent-cell clearance 9, 20, 81. Thus, senescence serves as a physiological barrier to oncogenesis.

With advancing age, two factors conspire to weaken this barrier. First, cells accrue senescence-inducing insults—telomere attrition, oxidative damage, and oncogenic mutations—at a higher frequency 53, 64, 82. Second, immunosenescence compromises surveillance: macrophage phagocytic capacity wanes, antigen-presenting cell function declines, naïve T-cell output diminishes, and T-cell receptor diversity contracts 48, 75. As a result, the presence of abundant senescent cells becomes a chronic feature of elderly people. Their chronic SASP secretion fosters a pro-tumor microenvironment by sustaining inflammation, promoting malignant transformation, suppressing immune clearance, and remodeling local stroma 44, 45.

### 3.2. Senescence and Cancer After Tumor Formation

Within established tumors, therapy-induced senescence (TIS) is prevalent 63. DNA-damaging chemotherapies and radiotherapy induce senescence in malignant cells 83-85. TIS was also observed in breast cancer, Ewing sarcoma, and neuroblastoma following treatment with CDK4/6 inhibitors 86, or in lung cancers and pancreatic cancers following treatment with MEK and CDK4/6 inhibitors 11, 87-90.

Senescent tumor cells do undergo growth arrest comparable to that of normal senescent cells. In various cancer models, senescent tumor cells uniformly exhibit cell-cycle arrest or markedly reduced proliferation 91. Does this mean that senescence of tumor cells facilitates effective tumor suppression? Indeed, therapy-induced senescent tumor cells can attract NK cells and DCs into tumor sites via the upregulation of MHC-I and IL-15/IL-15RA complex 7, 9. In lymphoma models with TIS, NK cells accumulate with enhanced response to tumor cells 92. Similarly, in another metastatic melanoma model with OIS, the senescence-induced infiltration of myeloid cells inhibited tumor growth 93. Preclinical evidence has also shown that therapy-induced senescent tumor cells induce complement activation and increase C3 expression 10. It seems that senescence brings hope to tumor suppression.

However, senescent tumor cells may paradoxically fuel disease progression. First, the growth arrest of senescent tumor cells is not stable. The re-entry into the cell cycle of therapy-induced or oncogene-induced senescent tumor cells has been demonstrated in mice and patients with breast cancers, colorectal cancers, and acute myelogenous leukemia 63. Mechanistically, increasing replication stress and DNA damage leads to genomic instability of oncogene-induced senescent tumor cells, enabling escape from growth arrest through various mutations 94. Therapy-induced senescent tumor cells, on the other hand, escape from cell-cycle arrest through multiple mechanisms, including metabolic reprogramming, chromatin remodeling, and signaling pathway rewiring 94. Second, preclinical and clinical observations have suggested that TIS may be detrimental. TIS has been associated with chemotherapy-induced cardiotoxicity, peripheral neuropathy, and ovarian damage in mice 16. Using four immunohistochemical markers, including lipofuscin, p16^INK4a^, p21^WAF1/Cip1^, and Ki67, researchers have found that the tumoral senescence signature significantly affected overall survival (OS) in 155 NSCLC patients 95. Single-cell analysis also revealed worse prognosis in patients with higher senescence signature 96. Third, senescent tumor cells foster an immunosuppressive tumor microenvironment. A higher senescence signature correlates with increased crosstalk between tumor cells and immune cells 96. This is not only attributed to SASP factors secretion, but also to the metabolic alterations of senescent tumor cells. While senescence-associated secretory phenotype (SASP) factors critically establish a protumoral TME, these will be addressed subsequently. Similar to non-malignant senescent cells, senescent tumor cells exhibit enhanced glycolysis 55. Such a metabolic shift not only promotes tumor invasion but also exacerbates the Warburg effect, driving lactate accumulation that impairs T cell and macrophage function 55, 97. Senescent tumor cells further display increased lipid uptake and diminished catabolism 55, 56, alterations that correlate with poorer clinical prognosis and immunotherapy resistance in cancer patients 98, 99. Cellular senescence additionally heightens tumor cell dependence on glutamine metabolism, facilitating cell cycle re-entry 100, 101. Notably, myeloid-derived suppressor cells (MDSCs) within the TME acquire mitochondrial DNA (mtDNA) released by senescent tumor cells, reinforcing their immunosuppressive activity 102. Collectively, this evidence underscores the therapeutic potential of ablating senescent cells. Consequently, senescence-targeting strategies in oncology broadly fall into two categories: inducing senescence to potentiate immune-mediated clearance or eliminating senescent cells to mitigate their chronic protumoral effects on the TME, which will be discussed in the following section.

## 4. Innate Immunity Senescence: From Bad to Worse?

Innate immunity acts as the first line of defense against pathogens. Recently, interest has grown in the role of innate immune cells within the TME 103, 104. Our focus will be on their induction, functional and phenotypic changes, and contributions to tumor progression.

### 4.1. Neutrophils

Neutrophils, pivotal components of the innate immune response, primarily originate from the bone marrow (BM) 105. Although neutrophils are short-lived, they can undergo senescence with functional consequences. One reason for their prolonged survival is impaired GM-CSF-induced apoptosis 106. In addition to physiological aging, it has been found that apolipoprotein E (APOE) secreted by tumor cells induces a subset of senescent neutrophils expressing the triggering receptor expressed on myeloid cells 2 (TREM2), which correlates with poor prognosis 107. Patients with breast cancer receiving chemotherapy harbor highly senescent neutrophils 17. This indicates that both tumors and cancer therapies can induce the senescence of neutrophils. Neutrophils become dysfunctional in killing microbes. Although neutrophil counts remain stable with age 108, 109, their defense against infection declines 110, 111. However, this does not necessarily mean that senescent neutrophils are dysfunctional within the TME. We will separately discuss the effects of senescence on the anti-tumor and pro-tumor functions of neutrophils.

Senescent neutrophils are defined as CXCR4^+^CD62L^low^ neutrophils 36, 112, 113. Neutrophils can exert antitumoral effects through various mechanisms 114, which can be potentially influenced by senescence. First, neutrophils can kill tumor cells opsonized with IgA or IgG via Fcγ- or Fcα-receptors 115, a process known as antibody-dependent cellular cytotoxicity (ADCC). Diminished Fcγ-mediated ADCC has been observed in senescent human neutrophils in both sexes, resulting from impaired free radical production 116. However, FcαR1 (CD89) is the principal receptor mediating neutrophil cytotoxicity against cancer cells 117, 118; therefore, it cannot yet be concluded that the overall ADCC capacity is reduced in senescent neutrophils. Neutrophils also exert antitumoral functions by secreting ROS and neutrophil extracellular traps (NETs) in certain scenarios. These functions will be focused on below. Moreover, neutrophils have been demonstrated to acquire antigen-presenting capabilities, bridging innate and adaptive immunity in lung cancer 119, 120. Their potential as antigen-presenting cells is further supported by recent profiling 121. However, further investigation is required to understand the influence of aging on this capacity. Overall, there is not yet sufficient evidence to definitively assess the impact of senescent neutrophils on tumor development. This may be due to the relatively recent association of neutrophils with tumors. Continued efforts are necessary to unravel the complexities surrounding neutrophil senescence.

**Figure 1:**
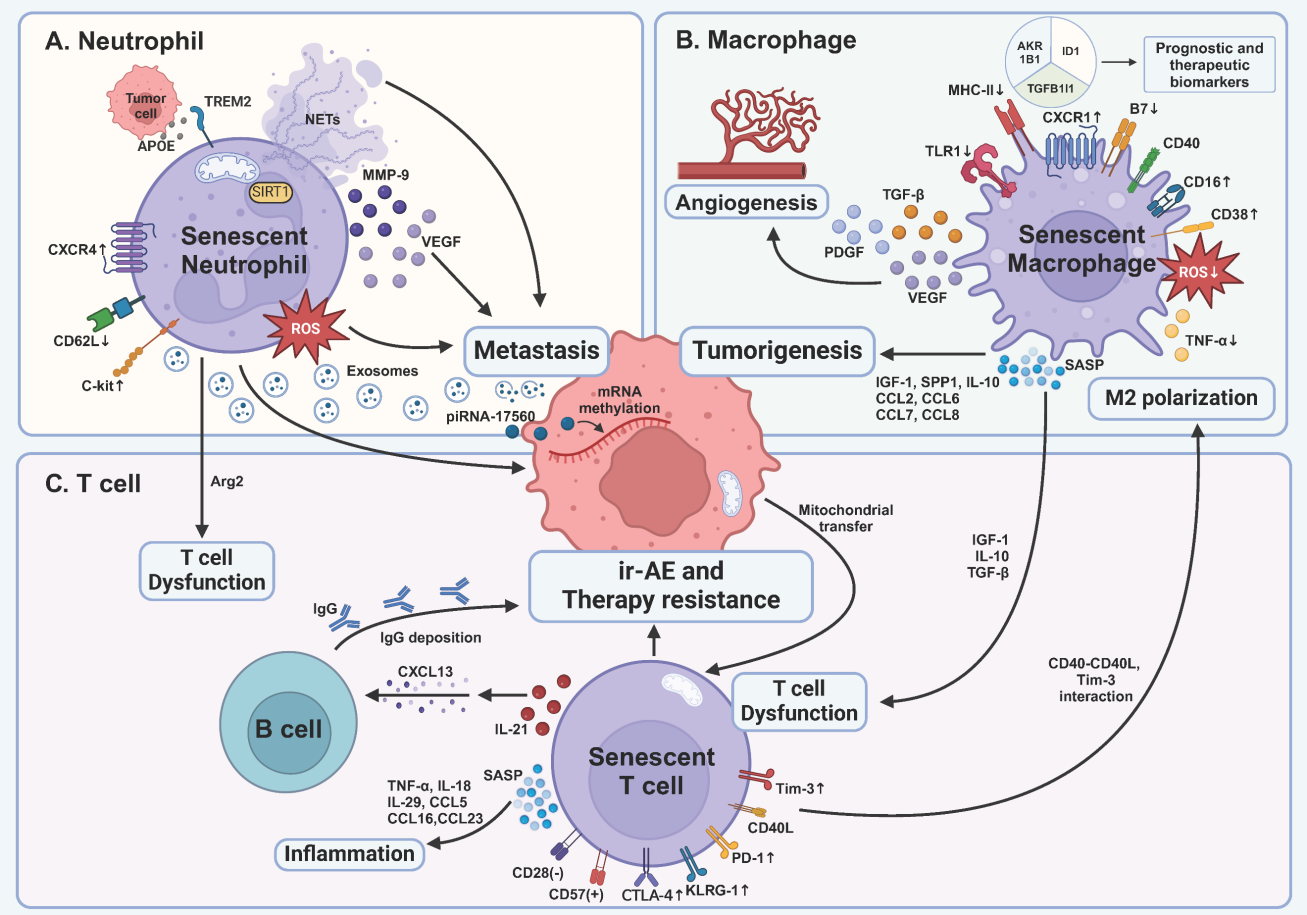
** Impact of senescent immune cells on tumor development and treatment within the tumor microenvironment. A** TREM2-expressing senescent neutrophils are induced by APOE secreted by prostate tumor cells, correlating with a poor prognosis. Senescent neutrophils promote cancer metastasis via distinct pathways, including ROS, mitochondria-dependent NETs, and cytokines. They also produce exosomes containing piRNA-17560, which causes chemotherapy resistance by RNA methylation of tumor cells. Senescent neutrophils lead to T cell dysfunction by Arg2 production. **B** Senescent macrophages' capability of killing tumor cells is inhibited, proven by decreased expression of MHC-II, B7, and impaired production of ROS and TNF-α. Senescent macrophages are another main force of SASP factors, leading to early tumorigenesis, angiogenesis, and immunosuppression. **C** Senescent T cells become dysfunctional as demonstrated by the expression of inhibitory receptors, including PD-1, Tim-3, and CTLA-4, and inhibitory SASP factors produced by senescent macrophages. In turn, senescent T cells enhance M2 polarization through CD40L and Tim-3 interaction. Moreover, senescent T cells secrete SASP factors to cause age-associated inflammation and deteriorate immune cell-related adverse events of ICB via IL-21-CXCL13-B cell-IgG axis. TNF-α, tumor necrosis factor-α; IL, interleukin; CTLA-4, cytotoxic T lymphocyte-associated antigen-4; Tim-3, T cell immunoglobulin and mucin domain-containing protein 3; PD-1, programmed death 1; KLRG-1, killer cell lectin-like receptor subfamily G 1; CXCL, C-X-C motif ligand; CCL, C-C chemokine motif ligand; SASP, senescence-associated secretory phenotype; ir-AE, immune cell-related adverse event; IGF-1, insulin-like growth factor 1; TGF-β, transforming growth factor-β; piRNA, PIWI-interacting RNA; NETs, neutrophil extracellular traps; TREM2, triggering receptor expressed on myeloid cells 2; APOE, apolipoprotein E; Arg2, arginase 2; SIRT1, silent mating type information regulation 2 homolog-1; MMP-9, matrix metallopeptidase 9; VEGF, vascular endothelial growth factor; ROS, reactive oxygen species; SPP1, secreted phosphoprotein 1; PDGF, platelet derived growth factor; TLR1, Toll-like receptor 1; MHC-II, major histocompatibility complex II. This figure was created with BioRender (https://biorender.com/).

Neutrophils exert a protumoral effect throughout the development of tumors **(Figure 1A)**. ROS, though observed to kill tumor cells in some research 122, has been demonstrated to promote chronic inflammation and carcinogenesis via nitric oxide (NO) production 123 and cause severe T cell immunosuppression 124. Given the elevated ROS levels in the elderly group 125, this increase may further promote tumorigenesis. NETs represent another age-associated mechanism that contributes to tumor promotion. NETs consist of DNA, histones, neutrophil elastase, matrix metalloproteinases, etc. 126. The impact of NETs on the tumor is complex. Certain components of NETs, including myeloperoxidase and defensins, can directly kill tumor cells 114, 126. The DNA structure of NETs is capable of capturing tumor cells, thereby preventing tumor metastasis 114. However, NETs can facilitate tumor proliferation, invasion, angiogenesis, and the formation of immunosuppressive TME 126. Therefore, it can be hypothesized that impaired NETs function in senescent neutrophils 127 may attenuate the aforementioned process, yet their overall impact on tumor development remains uncertain. Furthermore, neutrophils facilitate tumor metastasis **(Table 1)** 36, 113. Adoptive transfer of a subset of CXCR4^high^CD62L^low^ senescent neutrophils promotes tumor metastasis of breast and melanoma cancer cells to the liver 113. Accumulation of CXCR4^+^CD62L^low^ senescent neutrophils has also been found in the lung premetastatic niche at early stages of breast cancers, characterized by the expression of a specialized transcription factor SIRT1 36, 128. Finally, senescent neutrophils promote resistance to chemotherapy 17. Senescent neutrophil-derived exosomal piRNA-17560 stimulates the expression of fat mass and obesity-associated protein (FTO) in breast cancer cells, leading to chemoresistance and epithelial-mesenchymal transition (EMT) 17. Altogether, neutrophil senescence favors tumor progression, making senescent neutrophils a potential therapeutic target.

Indeed, emerging efforts to target neutrophils in cancer therapy are showing promise 114, although the influence of senescent neutrophils on these therapies remains unclear. Interestingly, researchers have recently trained neutrophils to eliminate tumor cells in a ROS-dependent manner 129, highlighting the ROS's potential in defending against tumors. Though increased levels of ROS have been found in older populations 125, in patients with breast cancers, resistance to chemotherapy has been attributed to senescent neutrophils 17. The efficacy of such approaches in elderly patients requires further exploration.

### 4.2. Macrophages

Macrophages are critical TME components, categorized as classically activated M1 macrophages and alternatively activated M2 macrophages based on their activation pathways 130. M1 macrophages, predominantly antitumoral, are identified by CD14^high^CD16^-^MHC-II^high^ expression, while M2 macrophages, which are protumoral, exhibit CD14^low^CD16^high^ MHC-II^low^ expression 130, 131. Senescent macrophages comprise a heterogeneous subset characterized by elevated CD38 expression 132. The senescence of macrophages can be induced by tumors. In a glioblastoma model, the 8B cells induced a senescence-like state of macrophages by the production of IL-6 133, typical components of SASP 8. This subset of macrophages, similar to M2 macrophages, produced high levels of Arginase-1 and inhibited T cell function within the TME 133, 134. Radiotherapy has also been found to induce senescence of myeloid cells in MC38 colon cancer models 19.

M1 macrophages are activated by Th1 cells or IFN-γ and kill tumor cells with mechanisms similar to those employed during infections, including ROS, lysosomal enzymes, and NO. Recruited by CD4^+^ T cells, M1 macrophages acquire the ability to eliminate pre-malignant senescent hepatocytes 20, thereby preventing tumor initiation. They also serve as antigen-presenting cells (APCs) to activate adaptive immunity. However, the antitumoral capacity of senescent M1 macrophages is compromised in several ways **(Figure 1B)**. Firstly, reductions in CD14^+^CD16^-^ macrophages, representing M1 subsets, have been observed in both aged humans and mouse models in the peripheral blood 33, 34. Using single-cell techniques, the M2 expansion in aged humans was also supported 135. In liver models with chronic damage, however, p53-expressing senescent hepatic satellite cells have been proved to polarize M2 subsets into M1 subsets 22, indicating the number of M1 macrophages may be organ-dependent. Secondly, the production of cytokines such as TNF-α, as well as the expression of TLR1, was impaired 33. The underlying mechanisms remain to be elucidated. Additionally, reduced ROS production in senescent macrophages was observed 8, weakening tumor immune surveillance. Furthermore, decreased expression of MHC-II and B7 costimulators in senescent macrophages indicates a diminished response to vaccination 136, 137. These findings demonstrate significant impairments in the antitumoral functions of senescent M1 macrophages.

M2 macrophages, driven by Th2 cells or Tregs, promote tumor growth through various mechanisms **(Figure 1B)**. As previously mentioned, increased M2 subsets are observed in elderly humans and mouse models 33, 34, 135. The accumulation of senescent macrophages further promotes tumor progression 23, 138. Prieto *et al.* have found that senescent alveolar macrophages expressing p16^INK4a^ and Cxcr1 increased in the lungs with aging in human and Kras-driven mice models, and their removal attenuated the tumor development 138. These studies underscore the significant role of senescent macrophages in early tumor initiation. M2 macrophages further promote tumor progression by inhibiting adaptive immunity and NK cells through the production of IL-10, TGF-β, and the expression of PD-L1 130. In aged mice, senescent macrophages produce elevated levels of IL-10 in the lungs, further suppressing the IL-12 axis, which is crucial for NK cell functionality 139. In an MC38 colon cancer model, radiotherapy-induced senescent M2 macrophages were sufficient to inhibit T cell functionality. The clearance of senescent cells reversed the proliferation of T cells, suggesting that senescence may foster an immunosuppressive TME regulated by M2 subsets 19. Finally, M2 macrophages promote angiogenesis through the production of VEGF, PDGF, and TGF-β 134, although the anti-angiogenic effect of senescent macrophages is compromised by the loss of Fas ligand (FasL) 140. Overall, these findings suggest that senescence makes macrophages more likely to promote tumor progression.

### 4.3. MDSCs

Myeloid-derived suppressor cells (MDSCs) comprise a heterogeneous group of myeloid progenitor cells and immature myeloid cells (IMCs) 141. They are categorized into monocytic (Mo-MDSCs) and polymorphonuclear MDSCs (PMN-MDSCs) 142, analogous to macrophages and neutrophils, respectively. MDSCs suppress tumor immunity through various mechanisms 141 while aging further enhances these immunosuppressive effects. First, aged mouse models exhibit expanded MDSC populations that produces IL-6 relevant to inflammaging 143-145. In the bone marrow of aged mice, MDSCs make up the majority of the NF-κB-expressing cells, suggesting NF-κB's role in their increase 145. Secondly, with aging, SASP factors can enhance the proliferation and functionality of MDSCs 146. p16^Ink4a^ and p21^Cip1/Waf1^ are highly expressed in Mo-MDSCs and stimulate CX3CR1 chemokine receptor expression, leading to the accumulation of Mo-MDSCs at tumor sites 147. Third, single-cell analysis revealed that, in the high-senescence-signature group, malignant cells exhibited a greater degree of interaction with MDSCs across many human cancers, indicating an enhanced immunosuppressive capacity of MDSCs 96. This evidence strongly suggests that aging reinforces the immunosuppressive role of MDSCs.

### 4.4. NK cells

NK cells are crucial for immune surveillance against cancers, consisting of immature CD3^-^CD56^bright^ NK cells and mature CD3^-^CD56^dim^ NK cells, with the latter accounting for the majority 148. Mature NK cells directly eliminate tumor cells via the production of perforin and granzyme B, or expression of FasL and TNF-related apoptosis-inducing ligand (TRAIL) 148, while immature NK cells contribute to tumor cell elimination by producing cytokines such as IFN-γ, TNF-α, and GM-CSF 149. Furthermore, NK cells play a critical role in neutralizing senescent cells, thereby preventing early tumorigenesis 8. For example, uterine NK cells clear senescent decidual cells following the induction of IL-15 150. Senescence impacts both subsets of NK cells. In a study examining changes in aged NK cells, an increase in NK cell numbers with age was observed in 11 out of 13 studies 151. The absolute number of immature CD3^-^CD56^bright^ NK cells has been shown to decrease with aging 152-154. Additionally, the response of NK cells to IL-2 was impaired 152, and their ability to produce IFN-γ and IL-8 was significantly inhibited 155, 156. In mature CD3^-^CD56^dim^ NK cells, age-related declines in perforin lead to reduced NK cell cytotoxicity (NKCC) 153. Additionally, the expression of NK cell activating receptors like NKp30 and NKp46 was reduced in elderly groups 154. Consistent with the reduction in NKCC, compromised tumor immunosurveillance of senescent NK cells against acute myeloid leukemia has been found 35. Overall, this evidence suggests that the senescence of NK cells leads to a diminished antitumoral effect.

### 4.5. Dendritic cells

Dendritic cells (DCs), as the quintessential APCs, play a critical role in bridging innate and adaptive immunity. DCs are classified as conventional DCs (cDC1 and cDC2) or plasmacytoid DCs (pDCs). cDC1 and cDC2 are respectively tasked with antigen presentation to CD8^+^ T cells via MHC-I and CD4^+^ T cells via MHC-II, while pDCs are dedicated to antiviral and antitumor immunity through the production of type I interferons 157. Aging impacts both cDCs and pDCs through several mechanisms. For cDCs, their absolute number remains unchanged with aging 37, and research has reported a diminished capacity for phagocytosis, migration, and T cell stimulation 37, 38. In mouse models with B16-ovalbumin (OVA) melanomas, senescent DCs failed to effectively stimulate T cells due to defective CCR7 signaling, despite an unchanged capacity of antigen presentation 38, which led to tumor progression. In aged humans, a decreased expression of MHC peptide and CD40 in cDCs was observed, subsequently impairing CD4^+^ T cell expansion 39. In pDCs, impaired production of type I and III interferon has been observed, resulting in reduced CD8^+^ T cell cytotoxicity 158. Moreover, NK cells were unable to activate and eradicate lymphoma tumor cells due to a deficiency in IL-15, IL-18, and IFN-α production by pDCs 159. Therefore, both DC subsets exhibit a functional decline in tumor immunity during aging.

## 5. Adaptive Immunity Senescence: The Main Force of Immunosenescence

Adaptive immunity plays a central role in tumor defense, with CD8^+^ cytotoxic T lymphocytes (CTLs) serving as the primary effector cells. Besides, CD4^+^ T cells, regulatory T cells (Tregs), and B cells all participate in the interaction of tumors and the TME. Thus, exploring the senescence of adaptive immunity is essential in discussions of immunosenescence and tumor progression. Our focus will primarily be on the role of senescent T cells within the TME, with a brief overview of senescent B cells, whose role in the TME remains less defined.

### 5.1. CD8^+^ T and CD4^+^ T Cells

Following cross-presentation and costimulation primarily by cDC1 in secondary lymphoid organs, naïve CD8^+^ T cells become activated and migrate to tumor sites, where they directly eliminate tumor cells through perforin/granzyme-mediated or FAS/FASL-mediated cytotoxicity. CD4^+^ T cells, particularly Th1 cells, enhance antitumor immune responses by augmenting CD8^+^ T cell activity and activating M1 macrophages by producing IFN-γ 160. Like other innate immune cells, both CD8^+^ and CD4^+^ T cells can eliminate senescent cells 9, 20, 81. Oncogene-induced pre-malignant senescent hepatocytes were cleared by macrophages, which were recruited by CD4^+^ T cells through the secretion of SASP 20. Furthermore, senescent cancer cells are highly immunogenic, facilitating their recognition and elimination by DCs and CD8^+^ T cells 9. T cells are crucial in the clearance of senescent cells.

T cells are the most extensively studied immune cells in the context of immunosenescence. Multiple pathways contribute to T cell senescence, with p38 and p53 being the most studied 4. Senescent CD8^+^ T cells were induced in LCMV-infected mice and aged CMV-infected patients 161, 162, characterized by expression of killer cell lectin-like receptor subfamily G 1 (KLRG-1) and impaired proliferation 162. TIS is also observed in T cells. In non-small cell lung cancer (NSCLC) patients, chemotherapy induces T cell senescence 163. Lately, researchers have found that chemoradiotherapy induced senescence of CD8^+^ T cells in human cervical cancers 14. Mechanistically, concurrent chemoradiotherapy triggers expression of atypical chemokine receptor 2 (ACKR2) on tumor cells, thus increasing the production of TGF-β and driving T cell senescence 14. Peripheral phospholipids were also responsible for T cell senescence 164. Furthermore, in various cancers including breast cancers, melanomas, colon cancers, prostate cancers, ovarian cancers and head and neck cancers 165-167, tumor-derived immunoglobulin-like transcript 4 (ILT4) and PD-L1 in EVs reprogrammed lipid metabolism and induced CD4^+^ T cell senescence via MAPK ERK1/2 signaling, leading to tumor progression and a poor prognosis 165, 168. Tumor-T cell contact can activate cAMP pathways to trigger CD4^+^ T cell senescence, a process reversed by tumor cell TLR8 activation 166. Recently, emerging evidence indicates that tumor cells further promote T cell senescence via mitochondrial transfer 169. Mechanistically, T cells internalize tumor-derived mutated mtDNA, promoting cellular senescence and compromising effector functions and memory formation 169. These findings underscore the previously underappreciated role of mitochondrial dysfunction in driving T cell senescence.

Senescence affects T cells in several ways **(Figure 1C)**. First, regarding surface markers, senescent T cells are typically characterized as CD28^-^CD57^+^CD4^+^/CD8^+^ T cells 170-172, which is observed in many types of cancer, including lung cancer, ovarian cancer, head and neck cancer, and glioblastoma, as mentioned above 173-176. Additionally, senescent T cells possess an increased expression of Tim-3, KLRG-1, and re-expression of the naïve T cell marker CD45RA 177, 178. Expression of PD-1 and CTLA-4 was also observed in patients with acute myeloid leukemia (AML) and visceral adipose tissue of obese mice 179, 180, suggesting potential immunosuppression. Second, the cytotoxicity of senescent CD8^+^ T cells is reduced, as evidenced by lower levels of perforins and granzyme B 30, 31, 181, which leads to impaired antitumor immunity 181. In contrast, senescent CD4^+^ T cells maintain their cytotoxic potential, with unchanged levels of perforins and granzyme B 182. Third, senescent T cells acquire SASP, which is related to age-associated inflammation 183. However, its role within the TME remains unclear. Fourth, senescent T cells modulate monocytes/macrophages through upregulated surface markers Tim-3 and CD40L 177. This leads to the production of pro-inflammatory cytokines and angiogenic factors, including TNF, IL-1β, IL-6, MMP-9, VEGF-A, and IL-8 184. Interestingly, when co-cultured with senescent T cells, monocytes/macrophages exhibit increased CD16 expression, a characteristic of M2 macrophages 130, 131, 184. It can be hypothesized that senescent T cells promote the polarization of macrophages from M1 subsets to M2 subsets. Fifth, senescent T cells undergo metabolic reprogramming akin to that of senescent somatic cells, characterized by enhanced glycolysis, mitochondrial biogenesis, and upregulated lipid metabolism 185, 186. Accumulation of lipid droplets in these cells impairs effector functions and diminishes the efficacy of T-cell-based immunotherapies 187, while increased glycolytic flux further amplifies SASP secretion 185. Overall, the evidence suggests that T cell senescence promotes a shift towards an immunosuppressive TME.

Accurate discrimination between senescent and exhausted T-cell phenotypes is essential, as both states are marked by functional impairment and co-express inhibitory receptors such as PD-1 and CTLA-4 179, 180. First, exhausted T cells are induced by constant stimulation of antigen, including chronic infection and cancer 188, wherein naïve T cells exhibit impaired differentiation into effector/memory subsets. Instead, they progress through precursor exhausted to terminally exhausted states 188. Conversely, senescent T cells derive from effector or memory T cells 189. Second, senescent T cells are typically regarded as CD28^-^CD57^+^CD4^+^/CD8^+^ T cells. Early T cell exhaustion is identified by expression of PD-1, TCF-1, and low expression of EOMES, while terminal T cell exhaustion is identified by high expression of PD-1, EOMES, and loss of TCF-1 188. On the contrary, senescent T cells exhibit far lower levels of PD-1 and CTLA-4 compared to exhausted T cells 161. Third, while immune checkpoint blockade (ICB) can rejuvenate exhausted T cells, it has little effect on senescent T cells 177. This phenomenon can be attributed to the differential expression of inhibitory receptors 161. Currently, there are no viable approaches to reverse T cell senescence. Moreover, an optimal therapeutic effect from ICB requires the coreceptor CD28, which is absent in senescent T cells 190, 191. Fourth, senescent T cells display a highly differentiated phenotype marked by the loss of CD27 and CD28 4, whereas exhausted T cells can be categorized into several subsets based on their differentiation 192. Thus, T cell senescence appears to be an irreversible endpoint, whereas T cell exhaustion may represent a reversible process.

Clinically, both senescent CD4^+^ T and CD8^+^ T cells were associated with poor survival rates and immunotherapy response in cancer patients 193-195, indicating that they may pose a barrier to effective cancer therapies. In metastatic breast cancer, patients undergoing chemotherapy exhibited a correlation between the increased number of senescent CD28^-^CD57^+^ T cells and shorter progression-free survival (PFS) 196. This correlation may be due to the elevated levels of IL-6 and IL-10 196, yet the mechanisms by which senescent T cells impact chemotherapy outcomes remain unclear. Regarding ICB, though it has minimal effects on senescent T cells, T cell senescence has been correlated with a lack of ICB benefit in elderly patients with distinct cancers 18, 194. Furthermore, aged mice experienced more ICB-induced adverse events compared to young mice, mediated by the IL-21-CXCL13-auto-antibody axis in CD4^+^ T cells 40, highlighting senescence as a risk factor for ICB. Nonetheless, a multicenter study found that elderly patients with melanoma responded more efficiently to anti-PD-1 therapy 197. This paradoxical finding warrants further investigation. It may be that senescent tumor cells become more susceptible to T cell immunity following PD-1-PD-L1 interaction blockade 198. Together, senescent T cells become dysfunctional and contribute to an immunosuppressive TME, with their clinical implications necessitating further investigation.

### 5.2. Tregs

Regulatory T cells (Tregs), identified as CD4^+^FOXP3^+^CD25^high^ T cells, play an important role in regulating tumor immunity. Tregs suppress tumor immunity through five primary mechanisms 199. In addition, Tregs are capable of inducing immunosenescence 200, 201. Firstly, Tregs induce DNA damage in T cells via glucose competition, subsequently leading to T cell senescence via p38, ERK1/2, and STAT pathways 200, 201. Furthermore, a subset of Tregs, known as γδ regulatory T cells, can induce senescence of T cells and DCs in breast cancer models 202. Interestingly, similar to tumor cells, activation of TLR8 with TLR8 ligands has been found to inhibit Treg-induced senescence by abrogation of Treg activity 201. Tregs are also influenced by aging. Studies have demonstrated that aged mice exhibit increased numbers of Tregs and higher FOXP3 expression. This subset of Tregs produces elevated levels of IL-10 and suppresses T cells and DCs more effectively compared to their younger counterparts 203. Single-cell analysis similarly revealed that, in cancers exhibiting a high senescence signature, there was increased infiltration of regulatory T cells (Tregs), which facilitated immune evasion and consequently promoted tumor progression 96. Together, aged Tregs in the TME exhibit enhanced immunosuppressive capabilities.

### 5.3. B cells

Historically, B cells were considered minor contributors to tumor immunity, but recent studies have challenged this view 204. It is now clear that B cells contribute to antitumor immunity through multiple mechanisms. First, activated B cells differentiate into plasma cells that secrete antibodies. IgGs have been found to coat tumor cells, facilitating their internalization by DCs and subsequent T cell activation 205. Moreover, IgG-secreting B cells can inhibit cancer cell growth in early-stage NSCLC 206. Different from IgGs, IgAs eliminate tumor cells in ovarian cancers through transcytosis 207. Antibodies also indirectly enhance antitumor immunity through mechanisms including ADCC, antibody-dependent cellular phagocytosis (ADCP), and complement-dependent cytotoxicity (CDC) 204. Second, B cells have been observed presenting antigens to CD4^+^ T cells by MHC-II or cross-presenting antigens to CD8^+^ T cells by MHC-I, thereby activating T cells 204. Third, recent studies have demonstrated an association between B cells and tertiary lymphoid structures (TLSs) 208. TLSs are ectopic lymphoid organs beyond classical lymphoid organs, which develop at sites with chronic inflammation 209. The formation of TLSs relies on interactions between lymphoid tissue inducer cells (LTi cells) and stromal cells mediated by IL-7 and CXCL13 209. Subsequently, the production of VEGF, chemokines, and adhesion molecules facilitates the formation of high endothelial venules (HEVs) and the recruitment of lymphocytes 209. In injured kidney models, TLS formation was observed in aged but not young mice 210. Moreover, in aged tumor-bearing mice, IL-21 produced by CD4^+^ T cells induced CXCL13 secretion, thereby promoting TLS formation 40. This suggests that aging may drive the formation of TLSs. Mature TLSs create an environment that allows B cells to exert antitumor immunity, while B cells act as 'administrators' of these structures 204, 208. Interestingly, though TLSs are generally associated with a favorable prognosis in some cancers 204, in aged mice, TLSs promoted by CD4^+^ T cells led to ICB resistance 40. Currently, there is insufficient evidence to fully understand the impact of senescent TLSs on tumor development, highlighting the need for further research.

Aging influences B cells in several ways. Specifically, gut microbiota has been shown to induce B cell senescence 211. With aging, there is a significant decrease in B cells among peripheral blood mononuclear cells (PBMCs) due to reduced B lymphopoiesis in the bone marrow 212. Despite the decreased number of B cells, an age-related increase in IgG and IgA levels was observed in elderly groups, along with a decrease in IgD and IgM levels 213. Interestingly, these changes vary between genders 213. Moreover, inflammaging in aged groups leads to a reduction in B cell progenitors and an accumulation of oncogenic mutations 214. Although research focuses on the senescence of B cells, the link between senescent B cells and tumor immunity remains to be explored.

## 6. Stromal Senescence: The Supportive Structure of the TME

We have sequentially introduced the senescence of immune cells, but it is far from illuminating the complexities of the entire tumor microenvironment. The stroma is important in providing support and structure, promoting angiogenesis, regulating immunity, facilitating metastasis, and conferring chemoresistance during tumor progression, especially in cancers such as pancreatic cancer. It primarily comprises fibroblasts, endothelial cells, pericytes, and adipocytes, together with the extracellular matrix (ECM). Our discussion will focus primarily on the first two types of senescent stromal cells—fibroblasts and endothelial cells—and their roles within the TME.

### 6.1. Fibroblasts

Fibroblasts play a primary role in stromal formation, with cancer-associated fibroblasts (CAFs) receiving significant attention for their role in tumor progression. CAFs segregate into inflammatory CAFs (iCAFs) and myogenic CAFs (myCAFs), differentiated by their spatial localization 215. iCAFs are located away from tumor cells, whereas myCAFs are adjuvant to tumor sites 215. By secreting cytokines, chemokines, and other effector molecules, CAFs directly or indirectly remodel the TME, which involves crosstalk with immune cells, including polarization of immune cells, regulation of immunity, reduction of cytotoxic cytokines, upregulation of inhibitory receptors, and remodeling of the extracellular matrix (ECM) 216.

Various factors can induce fibroblast senescence. Radiotherapy causes DNA damage in fibroblasts, thereby triggering DDR and inducing senescence 217. Novel therapies, such as CDK4/6 inhibitors, induced senescence of fibroblasts through the downregulation of Mdm2 in a melanoma model 15. Histone deacetylase (HDAC) inhibitors, used to treat various tumors including T cell lymphoma and multiple myeloma, induce fibroblast senescence without DNA damage 218. Interestingly, obesity increases the levels of deoxycholic acid in the enterohepatic circulation, which in turn drives the senescence of hepatic stellate cells through DDR 219, highlighting obesity as a significant contributor to stromal senescence.

The tumor-promoting nature of senescent fibroblasts was first suggested by A. Krtolica *et al.* in 2001, demonstrating their role in tumorigenesis in aged organisms 29. Subsequent research has reinforced this finding. During tumor initiation, senescent fibroblasts promoted ovarian tumorigenesis, as evidenced by reduced tumor growth following the abrogation of the senescence program 220. Further studies indicate that IL-4 or IL-10-mediated Th2 immunity, which is activated by NF-κB, predisposes aged H-Ras-activated mice to squamous cell carcinoma compared to younger counterparts 221. MMP-3, secreted by senescent fibroblasts, leads to the dedifferentiation of premalignant epithelial cells, thereby increasing tumorigenesis risk 222. Moreover, stroma-derived osteopontin (OPN), a component of the ECM, facilitated premalignant cell growth in elderly groups 223. Interestingly, beyond endocrine effects, senescent fibroblast also stimulates neoplastic epithelial cell proliferation through the production of amphiregulin (AREG) in prostate models 224. Together, stromal senescence robustly induces tumorigenesis through multiple mechanisms.

**Figure 2:**
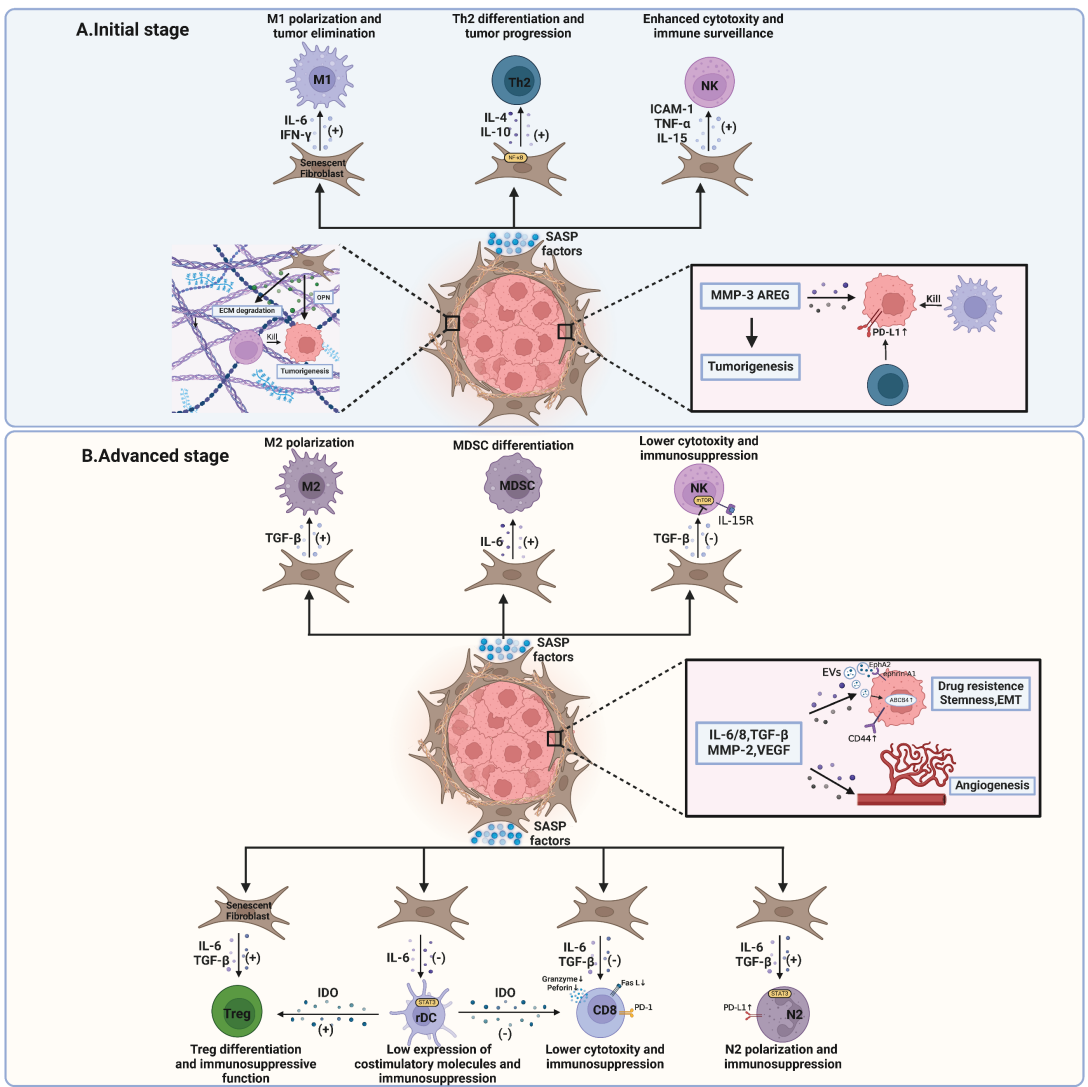
** Impacts of SASP produced by senescent fibroblasts within the tumor microenvironment. A** At the initial stage, senescent fibroblasts release SASP factors that encourage antitumoral immune responses. M1 macrophage polarization and NK cell-mediated cytotoxicity are bolstered by these factors. Additionally, ECM degradation by SASP factors facilitates enhanced immunosurveillance by NK cells. Conversely, other SASP factors may promote tumorigenesis through interactions with Th2 cells, which upregulate the expression of PD-L1 on tumor cells. ECM components like OPN can aid in tumor growth. **B** At advanced stages, SASP factors play a pivotal role in tumor progression. On one hand, they can induce cancer stemness, promote epithelial-mesenchymal transition (EMT), confer chemotherapy resistance, and stimulate angiogenesis. On the other hand, SASP factors from senescent fibroblasts contribute to an immunosuppressive microenvironment. This includes the recruitment of MDSCs, M2 and N2 polarization, inhibition of NK cell cytotoxicity, and Treg cell enhancement, which collectively inhibit effective anti-tumor immune responses. The interactions between PD-1 on T cells and PD-L1 on tumor cells further facilitate immune evasion by the tumor. MMP, matrix metalloproteinase; OPN, osteopontin; ECM, extracellular matrix; AREG, amphiregulin; ICAM-1, intercellular adhesion molecule-1; TNF-α, tumor necrosis factor-α; IL, interleukin; IFN-γ, interferon-γ; TGF-β, transforming growth factor-β; EVs, extracellular vesicles; VEGF, vascular endothelial growth factor; PD-1, programmed cell death protein 1; PD-L1, programmed cell death protein ligand 1; EMT, epithelial-mesenchymal transition; NK, natural killer; Th2, helper T cell 2; rDC, regulatory dendritic cell; IDO, indoleamine 2,3-dioxygenase; ABCB4, ATP-binding cassette subfamily B member 4; EphA2, erythropoietin-producing hepatocellular A2; ephrin-A1, recombinant human Ephrin A receptor 1. This figure was created with BioRender (https://biorender.com/).

Although senescent fibroblasts are often tumor-promoting, some studies indicate that during early stages, stromal senescence aids in recruiting immune cells **(Figure 2A)**, thereby facilitating the clearance of senescent cells and reducing cancer risk. In fibrotic murine livers, senescent HSCs exhibited increased ECM degradation, coupled with enhanced immune surveillance mediated by NK cells 225. In another murine liver fibrosis model, p53-induced senescence of HSCs resulted in macrophage polarization towards M1 subsets, mediated by SASP, including IL6 and IFN-γ 22. M1 macrophages, in turn, eliminate senescent HSCs, thereby limiting tumorigenesis 22. Though evidence has shown that senescent tumor cells can induce immune surveillance in several models, in addition to livers such as multiple myeloma and lung cancers 8, data on similar properties in senescent fibroblasts outside the liver are limited and warrant further investigation.

During advanced stages of tumors, senescent fibroblasts are pivotal in tumor invasion, metastasis, angiogenesis, and a poor prognosis **(Figure 2B)**. First, senescent fibroblast-derived MMP-2 and TGF-β induced keratinocyte invasion in squamous cell carcinoma models 226. Second, excessive IL-8 secreted by senescent fibroblasts enhanced invasion and metastasis in pancreatic cancer 227. The levels of IL-8 and stromal senescence, as represented by expression of p16^INK4a^, were associated with a poor prognosis of patients with pancreatic cancer 227. In another research, IL-6 and IL-8 induced EMT and stemness of breast cancer cells, as demonstrated by fibroblastoid morphology, increased expression of CD44, and enhanced self-renewal capabilities in tumor cells, making them more aggressive 228. Third, regarding angiogenesis, while early studies suggested reduced vascularization in aged tumor-bearing mice 229, subsequent research supports the idea that stromal senescence promotes vascularization via increased production of VEGF and TGF-β 27, 28. Fourth, extracellular vesicles (EVs), as heterogeneous types of membrane vesicles important for intracellular communication, were secreted by senescent fibroblasts 230, 231. Exosome, as a special category of EVs, was also found to be released in prostate cancers 232. Not only did EVs promote tumor proliferation through EphA2-ephrin-A1 interaction 231, but they also resulted in drug resistance via inducing expression of ATP-binding cassette subfamily B member 4 (ABCB4) 230. Interestingly, although traditional approaches emphasize inhibiting tumor angiogenesis 233, senescence-induced angiogenesis could be therapeutically employed 89, 90. Induced by MEK and CDK4/6 inhibitors trametinib and palbociclib (T/P), senescence successfully triggers SASP, including a series of pro-angiogenesis factors, which surprisingly enhances the therapeutic effect of chemotherapy and ICB in KRAS mutant pancreatic ductal adenocarcinoma (PDAC) 89, 90. This approach capitalizes on the desmoplastic nature of PDAC, which impedes drug delivery to tumor sites 234, 235. However, the viability of promoting angiogenesis through senescence in other tumor types remains uncertain. Finally, senescent fibroblasts upregulated gene expression relating to immune regulation and SASP, resulting in impaired CD8^+^ T cell cytotoxicity and poor responsiveness to immunotherapy and chemotherapy 236-239. The presence of senescent fibroblasts is correlated with a poor survival outcome using machine learning 238, 239.

### 6.2. Endothelial Cells

It is important to note that the stroma consists of more than just fibroblasts. Tumor-associated endothelial cells (ECs) also significantly impact the TME as a crucial stromal component. Analysis across various cancer types reveals that ECs exhibit the highest rate of cellular senescence among all cell types in the vascular compartment of cancers 240. In liver sinusoids, the majority of p16^INK4a^-expressing senescent cells are ECs 241. Indeed, ECs are particularly susceptible to senescence, being the first cell types affected by metabolites and senescence stimuli 46. Due to their critical location, various factors contribute to the senescence of ECs. Metabolites and hormones, including insulin, glucose, triglycerides, cholesterol, amino acids, ROS, endothelin I, and angiotensin II, can induce EC senescence. Senescent ECs, in turn, produce higher levels of ROS, endothelin I, and angiotensin II, creating a vicious cycle 46. Specifically, nitric oxide, crucial for vasodilation, is believed to attenuate EC senescence 47, 242. Conversely, the endothelial nitric oxide synthase (eNOS) is impaired in senescent ECs 243, indicating the interplay between NO and senescence. Cytokines such as TNF-α and TGF-β can induce senescence of ECs 47, 244. Moreover, like other cells, conventional cancer therapies 47, 245-247, targeted therapies including receptor tyrosine kinase inhibitors, VEGF inhibitors, and CDK4/6 inhibitors can all induce senescence of ECs 47, 248, 249. Interestingly, kisspeptin-10 (KP-10), a member of multifunctional peptides inhibiting metastasis of cancers, can induce endothelial senescence 250. In melanoma models, ECs exhibit upregulation of Krüppel-like factor 4 (KLF4), which induces senescence of ECs 13. This suggests indirect tumor cell involvement in EC senescence.

**Figure 3:**
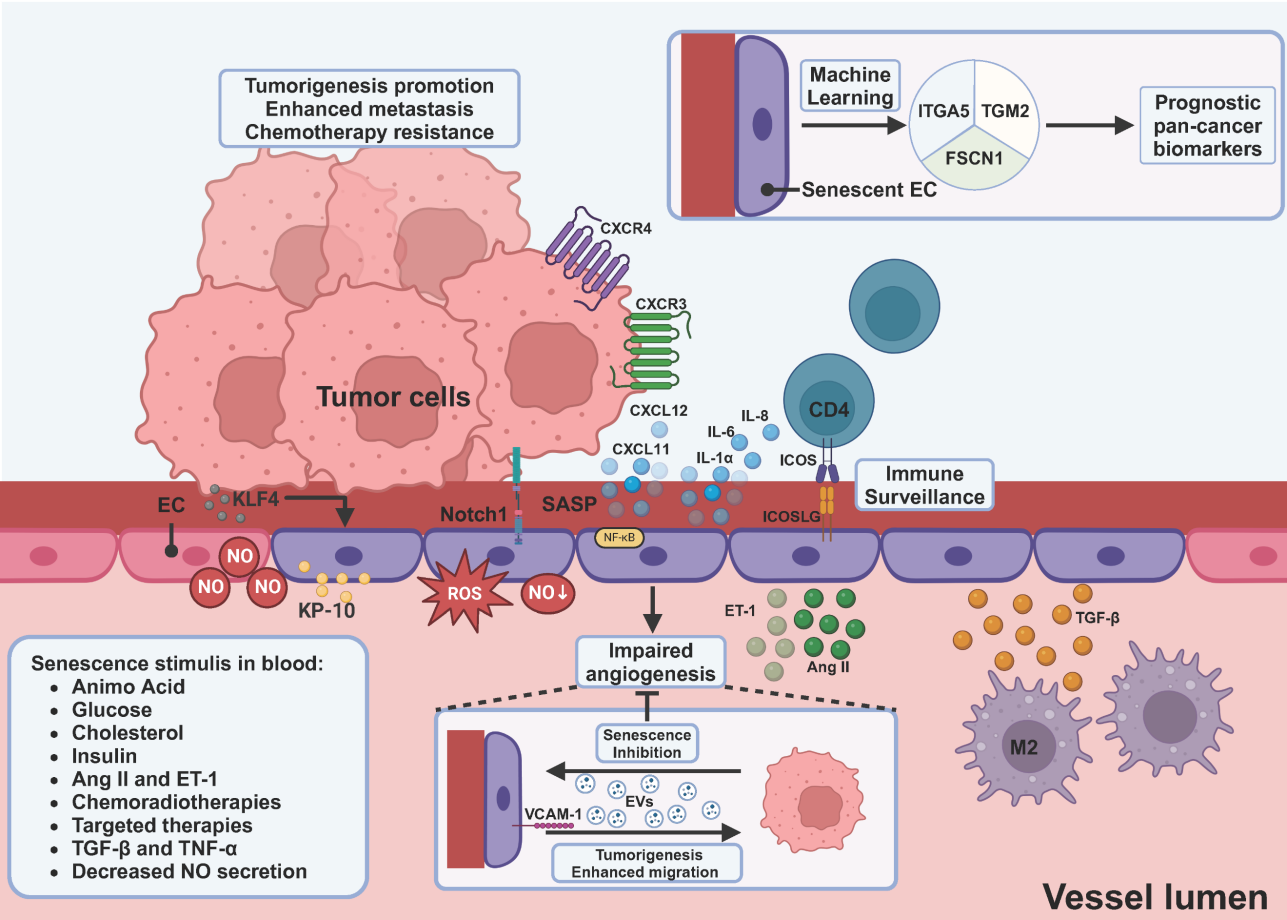
** Induction and impact of endothelial cell senescence within the tumor microenvironment.** Endothelial cells, as another important component of stroma, specifically become senescent when they encounter metabolites and hormones, including glucose, cholesterol, insulin, etc. M2-derived TGF-β can be another source of induction. Senescent endothelial cells produce increased levels of angiotensin II, endothelin 1, ROS, and decreased levels of NO, which in turn induce senescence of endothelial cells. Tumor-secreting KP-10 and upregulation of KLF4 on ECs can induce senescence of ECs. SASP factors produced by senescent endothelial cells have dual effects. On one hand, they lead to self-immunosurveillance mediated by CD4^+^ T cells. On the other hand, CXCL12 and CXCL11 can promote tumor cell metastasis and resistance to chemotherapy. Tumor-derived EVs are able to inhibit senescence of ECs, thereby counteracting the impaired angiogenesis of senescent ECs. Moreover, senescent EC-derived EVs and upregulation of VCAM-1 can promote proliferation, and migration of tumor cells. Using machine learning, ITGA5, TGM2, and FSCN1 were screened to be the potential prognostic pan-cancer biomarkers. EC, endothelial cell; NO, nitro oxide; Ang II, angiotensin II; ET-1, endothelin 1; KLF4, Kruppel-like factor 4; KP-10, kisspeptin-10; CXCL, C-X-C motif ligand; CXCR, C-X-C motif receptor; ICOS, inducible T cell co-stimulator; ICOSLG, inducible T cell co-stimulator ligand; VCAM-1, vascular cell adhesion molecule-1; ITGA5, integrin subunit alpha 5; TGM2, transglutaminase 2; FSCN1, fascin actin-bundling protein 1. This figure was created with BioRender (https://biorender.com/).

Senescent ECs have a dual role in tumor development **(Figure 3)**. On one hand, senescent ECs induce self-elimination by immune surveillance to evade tumorigenesis 21, with impaired angiogenesis capacity demonstrated by reduced proliferation and VEGF levels 247, 251. The benefit of this for cancer patients remains to be determined. On the other hand, senescent ECs promote tumor metastasis and treatment resistance via secretion of SASP 12, 13, 246. Moreover, the sustained activity of Notch1 receptors is observed in senescent ECs, which further promotes cancer metastasis through the production of VCAM-1 249, 252. Interestingly, though impaired angiogenesis was observed in senescent ECs, tumor-derived EVs can inhibit the senescence of ECs, thereby counteracting such effects 253. Moreover, senescent EC-derived EVs can promote the proliferation and migration of tumor cells 253. The pro-inflammatory profile of senescent ECs offers potential for survival prognostication and immunotherapy efficacy prediction using machine learning 240, 254, promising avenues for targeting or using senescent ECs as biomarkers.

## 7. Role of SASP Within the TME: A Double-Edged Sword

In the previous section, we have detailed the senescent immune and stromal cells within the TME. Notably, SASP is increasingly recognized as a key mediator of cellular senescence. Earlier perspectives suggested that senescent cells acquire SASP only when cellular senescence is triggered by DNA damage or the DNA damage response (DDR) 54, 255. However, current research suggests that SASP induction is a complex process mediated by multiple pathways 8, 41. Four primary pathways are now identified as mediators of SASP induction: p53-p21/p16-Rb, DDR-NF-κB, p38 MAPK, as well as mTOR and cytoplasmic DNA-cGAS-STING pathways 8, 256. Additionally, SASP is regulated by epigenetic mechanisms and oxylipins, such as dihomo-15d-PGJ2 256. The heterogeneity of SASP is influenced by the cell type and the causes of senescence, with IL-6 and IL-8 being commonly identified SASP factors 8. In this section, we will concentrate on the dual role of SASP in tumor progression. Research indicates that SASP secretion is influenced by tissue type, cell type, and stage of progression. Specifically, SASP dynamics within the tumor microenvironment can be categorized into two distinct stages.

During tumor initiation, SASP factors help eliminate potential pre-malignant cells. Senescent hepatocytes contribute to tumor surveillance through SASP-mediated senescence surveillance 80, 257, which relies on the participation of immune cells. These two studies underscore the importance of timely senescence surveillance in the liver. This has also been demonstrated in other cancers, including lymphoma, melanoma, and osteosarcoma, where innate immunity-mediated clearance of senescent cells provides tumor-suppressive effects 8, 45. Specifically, senescent pre-malignant cells may give rise to cancer if not cleared promptly. Senescent fibroblasts within the TME, as previously described, also exhibit anti-tumor activity during the early stages of tumor development. An exception arises in KRAS-driven lung cancer, where senescent macrophage SASP unexpectedly promotes early tumorigenesis 23, underscoring the need for deeper investigation into immune-derived SASP.

In established tumors, SASP fosters invasion, metastasis, and neovascularization, which we have elaborated on in the section on senescent fibroblasts. Senescent ECs produce SASP factors fostering metastasis in breast cancer 12 and melanoma models 13 and contributing to chemotherapy resistance 246. Moreover, the immunosuppressive microenvironment created by SASP factors should be emphasized. IL-6 regulates both innate and adaptive immunity. In innate immunity, through IL-6-STAT3 signaling, HCC-derived CAFs activate and maintain PD-L1^+^ neutrophils, thus impairing T cell function via PD-1-PD-L1 interaction 258. What's more, HCC-derived CAFs secreted IL-6 to generate regulatory DCs, which contribute to the dysfunction of T cells and the promotion of Treg activity via indoleamine 2,3-dioxygenase (IDO) upregulation 259. CAF-derived IL-6 promotes the differentiation of monocytes into myeloid-derived suppressor cells (MDSCs), thereby mediating immune dysfunction, which has been observed in HCC 260, pancreatic cancer 261, and esophageal squamous cell carcinoma 262. The extracellular matrix secreted by senescent fibroblasts was also able to limit NK cell cytotoxicity 263. In adaptive immunity, CAFs can directly enhance Treg function while inhibiting T cell proliferation through IL-6 production 264. Meanwhile, TGF-β, as another component of SASP 54, also acts as a regulator in tumor immunity. TGF-β not only promotes the transformation of monocytes into M2 macrophages 216 but also induces N2 neutrophil polarization in HCC 265. Moreover, TGF-β blocks IL-15-induced activation of mTOR, which is essential for cytotoxicity and proliferation of NK cells 266. Suppression of TGF-β successfully abrogated metastases in two mouse models 266. TGF-β derived from CAFs also promotes both Th17 differentiation and the conversion of CD4^+^ naïve T cells into Tregs 267, 268 while inhibiting the production of perforin, granzyme B, FasL, and IFN-γ by CD8^+^ T cells 269. Collectively, SASP factors produced by senescent cells are broadly immunosuppressive in advanced stages of tumors.

## 8. Novel Therapies Targeting Senescence: Next Hope for Cancer Treatment?

**Figure 4:**
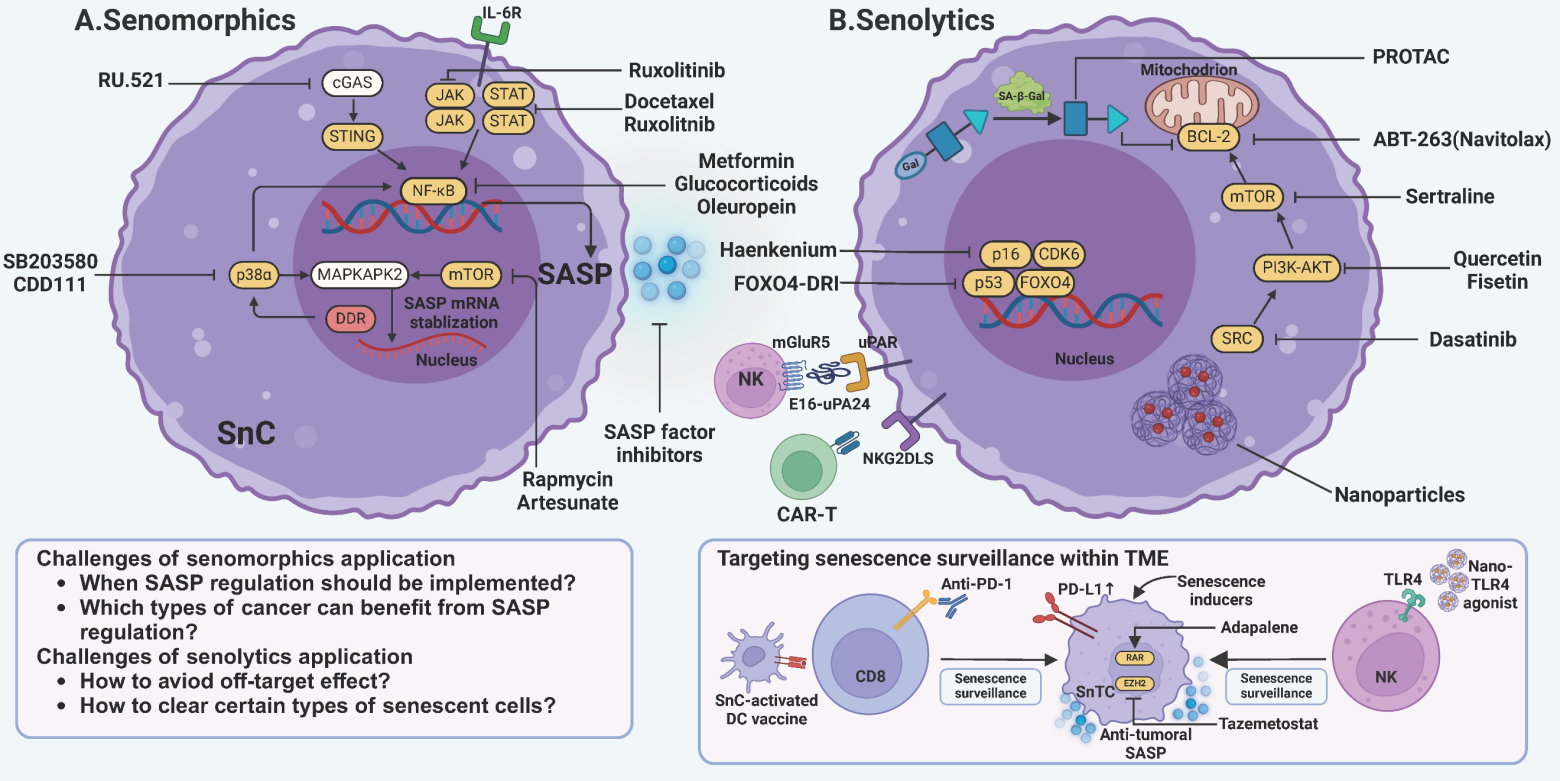
** Targeting senescence by modulation of SASP or clearance of senescent cells. A** One strategy to target senescence involves preventing senescent cells from producing tumor-promoting SASP factors. These drugs, termed senomorphics, primarily act on pathways such as cGAS-STING, JAK-STAT, and p38α-MAPKAPK2. Key mechanisms include NF-κB and mTOR to inhibit SASP production. Drugs such as metformin and rapamycin are among those used to modulate these pathways and mitigate the detrimental effects of SASP. **B** Another widely used strategy is to eliminate senescent cells with senolytics. The first-generation senolytics target anti-apoptotic pathways intrinsic to senescent cells, such as those involving BCL-2, PI3K-AKT, and mTOR. Second-generation senolytics find a new path by targeting specific surface markers on senescent cells. It utilizes innovative techniques like CAR-T cells, chimeric polypeptides, and vaccines. Notably, efforts are being made to enhance senescence surveillance mediated by T cells and NK cells. JAK, janus kinase; STAT, signal transducer and activator of transcription; NF-κB, nuclear factor-κB; cGAS, cyclic guanosine monophosphate-adenosine monophosphate synthase; STING, stimulator of interferon genes; mTOR, mammalian target of rapamycin; MAPKAPK2, mitogen-activated protein kinase-activated protein kinase 2; DDR, DNA damage response; BCL-2, B-cell lymphoma-2; PI3K, phosphatidylinositide 3-kinases; CAR-T, chimeric antigen receptor-T; GPNMB, glycoprotein nonmetastatic melanoma protein B; uPAR, urokinase-type plasminogen activator receptor; NKG2DLS, Natural killer group 2 member D ligands; SnC, senescent cell; SnTC, senescent tumor cell. This figure was created with BioRender (https://biorender.com/).

The advent of novel immunotherapies, including ICB, engineered chimeric antigen receptor (CAR) T cells, and cancer vaccines, has ushered in a new era in cancer treatment. Despite its success, ICB faces resistance driven by genetic and epigenetic aberrations in tumor cells, T cell exhaustion, cancer-associated fibroblasts (CAFs), and immunosuppressive mechanisms 270. Consequently, there is an urgent need to overcome these obstacles. Emerging evidence highlights the promising potential of targeting senescence to enhance the efficacy of ICB. These approaches fall into four categories—induction of senescence, regulation of SASP, clearance of senescence, and senescence reprogramming **(Figure 4)**.

### 8.1. Induction of Senescence

In the early stages of tumors, senescence exerts antitumoral effects through several mechanisms, including clearance of senescent cells, activation of tumor immunity, and promotion of proper angiogenesis, as discussed above 89, 90, 225, 270. Indeed, inducing senescence can improve the effect of cancer treatment **(Figure 4)**. T/P-induced senescence fosters the accumulation of CD8^+^ T cells, leading to increased sensitivity to ICB and chemotherapy in human PDAC models 89, 90. Interestingly, the desmoplastic nature of PDAC, which is typically resistant to drug treatment, was shown to benefit from T/P-induced SASP factor production, which promoted vascularization and improved drug delivery and chemotherapy response 89, 90. Additionally, induction of senescence stimulated the production of antitumoral SASP factors, leading to NK cell-mediated tumor clearance 271. EZH2 is a key gene regulating SASP secretion, whose blockade combined with ICB has successfully promoted the production of SASP chemokines, including CCL2 and CXCL9/10, leading to T cells and NK cells-mediated tumor immunity 11. Nanoparticles co-delivering senescence inducers and TLR4 agonists extend survival in PDAC by activating T cells and NK cells 272. Ali and JAK2 inhibitor ruxolitinib could also recruit T cells and NK cells within TME by inducing SASP secretion 273. DC vaccines loaded with senescent tumor antigens or PD-1 blockade further potentiate T cell responses 198, 274. Conclusively, T cells and NK cells are emerging as the primary force in eliminating senescent cells with the support of SASP. Beyond preclinical studies, clinical trials have explored inducing senescence with dexamethasone to re-sensitize response to ICB in patients with NSCLC (NCT04037462).

To harness the antitumor benefits of TIS while mitigating its deleterious immunosuppressive effects, two principles may guide clinical implementation. First, the temporal window for senolytic intervention is critical. Extended persistence of senescent cells within the TME fosters immunosuppression, angiogenesis, and metastatic niche formation as discussed above. Thus, senolytics should be deployed once TIS has maximally engaged immune-mediated tumor clearance but prior to the onset of a full-blown SASP or escape from growth arrest by senescent cells 275. Second, current senescence-inducing modalities—chemotherapy, radiotherapy, and kinase inhibitors—lack specificity and can inadvertently drive senescence in immune effector populations, exacerbating immune dysfunctions 14, 19. To obviate this, agents that selectively target tumor-cell senescence are required. For example, pharmacological inhibition of the replication origin kinase CDC7 induces senescence specifically in hepatocellular carcinoma cells without impairing normal immune cells 276. Similarly, the natural alkaloid tryptanthrin (TRYP) rapidly triggers senescence in liver cancer cells, arresting proliferation while sparing systemic immunity 277.

### 8.2. Regulation of SASP

Conversely, inhibiting the tumor-promoting SASP factors also emerges as a plausible alternative. **(Figure 4A)**. Drugs targeting SASP pathways are referred to as senomorphics. Key intervention points include transcriptional regulators, signal transduction cascades, metabolic nodes, and the SASP factors themselves 8, 41, 48. For instance, inhibiting the JAK2/STAT3 pathway, which is involved in SASP-associated tumor growth and chemoresistance, induces robust immune surveillance in Pten^null^ tumors with docetaxel-induced senescence 278. Targeting PTBP1 via RNA interference prevented the protumoral effects of SASP factors in tumor-bearing mice 279. NF-κB and mTOR have emerged as prominent targets for mitigating senescence 8, 92, 93. Metabolic reprogramming in senescent cells further dictates SASP output: in pancreatic cancer models, elevated NAD⁺ flux enhances NF-κB-dependent proinflammatory SASP 280, whereas inhibition of nicotinamide phosphoribosyltransferase (NAMPT)—the rate-limiting enzyme of the NAD⁺ salvage pathway—dampens SASP release and suppresses tumor growth 280. In the hepatic niche, loss of the gluconeogenic enzyme fructose-1,6-bisphosphatase 1 (FBP1) in hepatocytes triggers secondary senescence of hepatic stellate cells via HMGB1 signaling; neutralization of extracellular HMGB1 attenuates HSC-derived SASP and impairs tumor progression 281. Interestingly, metformin, a common medication for type II diabetes, can suppress NF-κB pathways 282. It can also inhibit T cell senescence while maintaining its cytotoxicity 283. Though metformin has shown potential in attenuating aging 284, NF-κB suppression unfortunately led to drug resistance and a poor prognosis in murine lymphoma and melanoma models 92, 93. Epidemiological studies suggest a decreased incidence of cancer in individuals receiving metformin 285, suggesting its potential role in cancer prevention rather than treatment.

The mTOR-MK2 pathway also plays a crucial role in SASP production 286-289. mTOR inhibitors like rapamycin reduce the secretion of tumor-promoting SASP factors 288, 289. In a phase IIa randomized controlled trial, the use of rapamycin enhanced the response to influenza vaccination 290, demonstrating its potential to boost immunity. Moreover, unlike other mTOR inhibitors, brief administration of rapamycin can produce a sustained anti-SASP effect, thereby reducing the risk of adverse events associated with long-term treatment 291. To date, clinical studies evaluating rapamycin's efficacy in targeting senescence remain in the early stages **(Table 2)** 292.

These findings raise the question: Can the regulation of SASP factors signify the next breakthrough in cancer therapy? The dual role of SASP in tumor development complicates its clinical application. Thus, balancing the antitumoral and protumoral effects of SASP factors is crucial. Based on current research on SASP so far, several key characteristics of SASP can be identified. The secretion of SASP factors is stage-dependent and tissue-dependent, which presents two major challenges. First, determining when SASP should be induced or inhibited remains a critical question. At present, it remains challenging to determine whether SASP is beneficial or detrimental for a particular patient. Nor can the exact point at which the role of SASP is reversed be identified. However, since many cancers are diagnosed at advanced stages, it may be more beneficial to inhibit the production of SASP factors to achieve improved clinical outcomes. Second, for certain tumor types, it remains unclear which strategy is optimal. The answer may lie in identifying which component of the SASP factors is dominant in regulating the TME as a whole. For instance, in pancreatic ductal adenocarcinoma (PDAC) models, pro-angiogenic factors produced by senescent cells can promote the formation of a more 'open' microenvironment, thereby enhancing the response to chemotherapy and immunotherapy 89, 90. While in lymphoma models. In lymphoma models, IL-6 produced by senescent endothelial cells (ECs) has been shown to protect tumor cells from chemotherapy 246. Overall, the future of regulating SASP as an effective cancer therapy is likely to be personalized.

### 8.3. Clearance of Senescence

Senescent cell clearance via senolytics, drugs that selectively ablate senescent cells, is a second therapeutic strategy **(Figure 4B)**. Unlike SASP inhibitors, senolytics remove the SASP source and can be dosed intermittently 297. Currently, there are two generations of senolytic drugs. First-generation senolytics target multiple antiapoptotic pathways (SCAPs) in senescent cells, such as BCL-2, SRC kinases, PI3K-AKT, etc. In contrast, targets of second-generation senolytics are discovered via high-throughput library screens, and include lysosome‑targeted agents, vaccine‑based approaches, nanoparticle delivery, and CAR‑T cell strategies 297.

Classic first-generation senolytics strategies include dasatinib (D) plus quercetin (Q) and navitoclax (ABT-263). D plus Q induces senescent cell death by inhibiting tyrosine kinase and PI3K signaling, respectively 298. The combination of D and Q has been observed to alleviate symptoms and increase survival rates in various age-related diseases, including postmenopausal osteoporosis 299, intervertebral disc degeneration 300, diabetic kidney disease 301, and SARS-CoV-2 302. D plus Q can indirectly suppress tumor development and metastasis by mitigating stromal senescence 303, 304. This effect is attributed to the inhibition of protumoral SASP secreted by senescent fibroblasts and stem cells 303, 304. Clinical trials (NCT04733534, NCT05724329, NCT06355037) are ongoing to determine whether D plus Q can be a viable approach to reverse chemoresistance or to improve survival as an effective and safe adjuvant therapy. ABT-263, one of the BCL-2 inhibitors, has demonstrated greater success in the context of cancer therapy, with the capacity to eliminate therapy-induced senescent cells in cancer models such as lung cancer, breast cancer, melanoma, ovarian cancer, and prostate cancer 45, 298, 305. In preclinical studies, ABT-263 reversed side effects associated with TIS, including bone marrow suppression, cardiac dysfunction, and cancer recurrence 306. Clinical studies combining ABT‑263 with chemotherapy are in progress **(Table 2)** 45, 293-296, 307. Finally, fisetin is another promising senolytic targeting senescence in cancers. Fisetin is extracted from vegetables and fruits, with a mechanism of action similar to that of quercetin 298, 308. In patients with small-cell lung cancers, fisetin successfully reversed the chemotherapy resistance induced by cellular senescence 309. Several phase II clinical trials (NCT04733534, NCT05595499, NCT06113016) are underway to evaluate its efficacy and safety targeting cancers.

Despite the promise, senolytics have drawbacks. First, patients receiving ABT-263 are at risk of developing thrombocytopenia and neutropenia 45, 297, raising concerns about its safety. Second, resistance to BCL inhibition in senescent tumor cells has been reported 310, 311, though efforts are underway to target mitochondrial apoptotic pathways or employ sensitizer proteins to restore sensitivity to senolysis 310, 312. Third, D plus Q failed to directly kill senescent cells and even exhibited tumor-promoting effects when used alone in animal HCC models due to the poor penetration in tumor sites 313, 314. The potential of D plus Q in cancer treatment may be realized through novel delivery approaches, such as extracellular vesicles and nanoparticles 314, 315. Fourth, the elimination of certain senescent cells may cause adverse consequences 241. For example, acute clearance of senescent ECs in livers will compromise blood-tissue barriers, potentially accelerating liver fibrosis 241. In light of these limitations, targeting specific markers to clear senescent cells, or eliminating certain types of senescent cells, has emerged as an alternative approach, namely the second-generation senolytics.

Second-generation senolytics exhibit enhanced target specificity, exploiting senescence-associated pathways through integrated immunotherapeutic strategies—including cancer vaccines, CAR-T cells, and antibody-drug conjugates (ADCs)—to achieve selective clearance **(Figure 4B)** 297. SA-β-Gal is overexpressed in senescent cells, which is the most commonly used senescence marker 11, 47. Not only can it be applied to specifically deliver ABT-263 316, but it can also be recognized by engineered proteolysis-targeting chimeras (PROTACs), thereby selectively eliminating senescent cells 317-321. Composed of a galactose (Gal) moiety, PROTACs like ARV-771 and MS999 can effectively clear senescent tumor cells without inducing significant adverse events 315, 318. Another PROTAC drug 753b targeted BCL-xL and BCL-2 dually to inhibit tumor progression 321. Moreover, new senescence markers are being found. For instance, urokinase-type plasminogen activator receptor (uPAR), a surface protein broadly expressed in T/P induced senescent cells, can be targeted by CAR-T cells, and its elimination improved the prognosis of mice with lung adenocarcinoma 87, 88, 322. Moreover, using a chimeric polypeptide, uPAR-expressing senescent cells can be cleared by NK cells 323. Natural killer group 2 member D ligands (NKG2DLs), another surface marker widely expressed in senescent cells, can be targeted by CAR-T cells safely 324. The effectiveness of this approach in cancer treatment requires further investigation. Notably, CAR-T cells, like conventional T cells, may also undergo senescence 48. First, children and young adults with B-ALL have benefited most from CAR-T therapy 325, and current clinical trials have yet to report great benefits in elderly patients (NCT05523661, NCT04537442, NCT05707273, NCT04300998). Second, research has observed increased expression of CD57 on CAR-T cells in the highly malignant glioblastoma multiforme models 326, and modulating p53 signaling pathways helps enhance CAR-T therapy in patients with chronic lymphocytic leukemia 327, suggesting the potential for CAR-T cell senescence. Finally, targeting metabolic dependencies of senescent tumor cells offers an additional avenue for senolytic intervention, given their heightened reliance on glycolysis and glutaminolysis for survival 55, 56. Indeed, inhibiting glucose uptake or metabolism induces apoptosis selectively in senescent tumor cells 328, while blockade of glutamine utilization suppresses their escape from growth arrest 100. A potential of mitochondrial-targeted therapy is also suggested. Mitochondrial physiology likewise serves both as a vulnerability and a biomarker for senolytic sensitivity 329-331. Specifically, pharmacologic inhibition of the TBK1-ATAD3A-Pink1 axis attenuates Pink1-mediated mitophagy, mitigates cellular senescence, and enhances chemotherapeutic efficacy 329. Moreover, mitochondrial dependence on BCL-XL and MCL-1 has emerged as a robust biomarker for forecasting senescent cell responsiveness to ABT-263 330, 331. Collectively, these findings position mitochondrial targeting at the forefront of next-generation anti-senescence therapies.

Based on the success of immunotherapy in cancer treatment, there is growing interest in targeting senescence to reverse the immunosuppressive microenvironment, thereby resensitizing immunotherapy. Senescent tumor cell clearance has been proven to reverse the immunotherapy resistance associated with the accumulation of senescent cells 19. Senolytics disrupted SASP-mediated PD-L1/TGF-β signaling axis and replenished intratumoral CD8^+^ T cells with restored granzyme B expression by normalizing TME arginine metabolism via arginase-1 suppression 19. Neutralization of senescent-cell-derived mtDNA reverses PMN-MDSC-mediated immunosuppression, enhances T-cell function, and potentiates chemotherapy 102, 169, 332. Beyond malignant cells, senescent immune and stromal populations are also amenable to targeting **(Table 3)**. Inhibition of cholesterol biosynthesis and lipid droplet formation prevents T-cell senescence and restores checkpoint-inhibitor efficacy 168, 187. CD153, highly expressed in senescent T cells, can be recognized and eliminated by the CD153-CpG vaccine in mice with obesity-induced senescence 333. One year after the first vaccine was invented, another seno-antigen glycoprotein nonmetastatic melanoma protein B (GPNMB) was screened via analysis of the transcriptome, and vaccination against GPNMB on senescent ECs was also effective in clearing senescent cells in mice with obesity-induced senescence 334. Moreover, the elimination of senescent fibroblasts with senolytics awakened T cells and NK cells-mediated tumor immunity and resensitized response to chemotherapy in breast cancers and pancreatic cancers 236, 237, 263. Senescent macrophages can also serve as a target, with their elimination through senolytics ameliorating early tumor growth and facilitating ICB-based immunotherapy 19, 23, 138. Together, second-generation senolytics hold promise for attenuating senescence. Further clinical trials are needed to determine their safety and efficacy as cancer therapies.

Finally, a therapeutic paradigm known as the “one-two punch therapy” has emerged, whereby induction of tumor-cell senescence is immediately followed by selective senolysis to maximize antitumor efficacy while limiting chronic SASP-driven toxicity 41, 43, 45. In TP53-mutated liver cancer, inhibition of the DNA-replication kinase CDC7 specifically induced senescence of liver cancer cells, while subsequent treatment with mTOR inhibitors sertraline markedly reduced tumor growth 276. The combination of 'one-two punch' therapy and immunotherapy has demonstrated potent inhibition of tumor growth in colorectal cancer and lung cancer 336, 337. Ongoing efforts are identifying inducers and senolytics with enhanced specificity for senescent tumor cells to improve the therapeutic index 277, 337, 338. Notably, perturbation of methionine catabolism precipitates DNA damage-mediated senescence in liver cancer cells, and follow-on senolytic therapy effectively attenuates hepatocarcinogenesis 58, suggesting that metabolic targeting may unlock a new frontier for precise, safe senescence induction.

### 8.4. Senescence Reprogramming

Finally, since T cell exhaustion is reversible by ICB 177, researchers are exploring methods to revert cellular senescence. Opinions once held that no viable strategy had been identified to achieve this reversal, but recently, approaches have emerged to reverse senescence 339, 340. Gu *et al.* showed that FBP1 suppression bypasses senescence in HCC progenitors, restoring their proliferative capacity 340.

Bi *et al.* demonstrated that exosomes derived from human embryonic stem cells and their miR-302b content can reverse cellular senescence by targeting key cell cycle inhibitors, leading to rejuvenation in aging mice without safety concerns 339. These two studies have unveiled the potential for reversing aging, raising anticipation for further research.

Moreover, the aged immune system can be reprogrammed to generate rejuvenated immune cells. First, one of the characteristics of natural immunosenescence is the involution of the thymus 48, 49, leading to reduced output of naive T cells. Efforts to rejuvenate the thymus have shown promise. Aged mice receiving IL-7 have shown enhanced adaptive immunity, as evidenced by lower viral load 341, while the thymostimulatory property of IL-21 was further demonstrated in the humanized mice model 342, 343. Gene modulation targeting Foxn1 can also partially rescue thymic involution and reduction of peripheral CD4^+^ T cells via exogenous FoxN1-cDNA 344, although recent research has indicated that Foxn1 overexpression does not prevent thymic involution 345. The second approach is implementing hematopoietic transplantation. Through intrathymic injection of hematopoietic progenitor cells from healthy mice, thymic reconstitution could be achieved in mice with severe combined immunodeficiency 346. Umbilical cord blood (UCB) can be an alternative source of HSCs 347. Finally, CD8^+^ T cells isolated from HIV patients can be reprogrammed to pluripotent stem cells, which subsequently re-differentiate into CD8^+^ T cells with enhanced cytotoxicity and proliferation 348.

## 9. Conclusion and Perspectives

In conclusion, senescence is a ubiquitous process affecting all components of the TME. In this review, we highlighted that senescence extends beyond chronological aging, representing the sum of diverse senescence triggers. Aging can be understood as the cumulative effect of senescence inducers. In clinical settings, conventional and novel cancer therapies, oncogene-induced senescence, and interactions within the TME are significant contributors 54, 83-85. This underscores the need for senescence studies to extend beyond just elderly populations.

Next, we have reviewed the properties of senescent immune cells in both innate and adaptive immunity, as well as the impact of SASP factors produced by senescent stromal cells. While adaptive and stromal senescence are well characterized, innate immune senescence in cancer remains understudied. This knowledge gap reflects the recent recognition of senescence in neutrophils and macrophages 50, 349. As increasing studies have elucidated the roles of neutrophils and macrophages in tumor immunity 114, 121, 130, 134, 350-352, it becomes imperative to investigate the controversial role of innate immunosenescence in tumors.

Finally, we outline existing therapies targeting senescence. Though great progress has been made in targeting SASP and senescent cells, their clinical application remains distant, as discussed in the corresponding section above. Moreover, barriers to the clinical implementation of senescence-targeted therapy can be partly attributed to the classification of senescent cells in human samples, since it is essential for understanding how senescence influences responses to cancer therapy and clinical outcomes, and to what extent senescence-targeting therapy can benefit from cancer treatment 63. Saleh *et al.* reviewed 21 studies that aimed to identify therapy-induced senescent cells in patient samples 16 and highlighted current limitations, including limited approaches for senescence detection, the challenge of obtaining cancer samples from patients who have not undergone chemotherapy, the requirement for freshly frozen tissue for SA-β-gal staining, and variability in baseline expression of senescence markers across different cancer samples 16.

To address these challenges, it is urgently necessary to find solutions for the following issues: (a)To identify reliable inducers of senescence. Numerous factors can induce senescence, but identifying the most suitable one for laboratory and clinical conditions is crucial. Chemotherapy or radiation-induced senescence is not suitable for all tumor types, especially for those treated with chemotherapy after surgery 16. Finding an inducer that works universally across cell types or matching various tumors with their viable inducers is essential. Typically, Scott W Lowe et al. have utilized T/P to induce senescence of pancreatic cancers and lung cancers 11, 87, 89. However, a stable inducer for research on immunosenescence is still lacking. (b)To standardize existing markers. So far, SenNet has recommended senescence markers across different tissues of humans and mice 353. Standard protocols exist for senescence research in vivo. According to the minimum information for cellular senescence experimentation in vivo (MICSE) published in 2024 354, markers used to detect senescent cells should include at least three markers of different properties of cellular senescence, at least one of which should be increased p16^INK4a^ or p21^Cip1/Waf1^ expression. However, no standard has been established for clinical trials since MICSE is not intended for clinical practice. In breast cancers, progress is being made toward standardizing senescence detection, including the establishment of baseline Lamin B1 expression and a three-marker signature approach to detect TIS, which involves downregulation of Lamin B1 and Ki-67 and upregulation of p16^INK4a^ 355, 356. (c)To discover emerging markers. Discovering specific markers will aid in understanding functional changes and targeting specific senescent cells. For instance, senescent cells can be isolated using flow cytometry by the differential presence of dipeptidyl peptidase 4 (DPP4) 357. Anti-DPP4 antibodies enable natural killer (NK) cell-mediated elimination of senescent cells, offering new perspectives on senescence-targeted therapy 357. CD153, differentially expressed in senescent T cells, could be applied as a vaccine to selectively clear senescent T cells in mice 333. Moreover, advances in technology, such as artificial intelligence, high-throughput sequencing, and single-cell sequencing, offer new opportunities for studying senescence. For instance, uPAR, as one of the targets of CAR-T, was discovered with RNA-sequencing 87, 88. Discovery of novel senolytics can now be achieved using machine learning 358. Whether these new markers can be the next-generation markers in clinical practices remains to be validated. (d) To create novel detection approaches. Machine learning and artificial intelligence are gaining popularity in detecting senescence 353, 359, although identifying specific types of senescent cells remains challenging. Recently, Zhou *et al.* have introduced a brand-new approach to specifically trace certain types of senescent cells 360. In this study, they generated pulse-chase tracing (Sn-pTracer), Cre-based tracing and ablation (Sn-cTracer), and gene manipulation combined with tracing (Sn-gTracer) to track p16^INK4a^ macrophages and ECs, thereby enabling the clearance of specific types of senescent cells 360. It is believed that targeting senescent cells will become a reliable cancer therapy in the near future.

## Figures and Tables

**Table 1:** Types of senescence and their roles within the tumor microenvironment

Type of senescence	Triggers	Role in TME	Mechanism	Senescent cell	Ref.
TIS	Chemotherapy Radiotherapy Targeted therapy	Anti-tumor	Immune surveillance by NK cells and macrophages	Tumor cell	7-9
			Complement activation	Tumor cell	10
			Recruitment of DCs and T cells	Tumor cell	7, 9
			Sensitization of chemotherapy and ICB in PDAC	Tumor cell	11
		Pro-tumor	Metastasis promotion	Tumor cell Fibroblast EC	8, 12, 13
			Invasion promotion	EC	12
			Stemness induction	Tumor cell	8
			Immunosuppression	CD8^+^ T cell Fibroblast	14-16
			Chemoresistance and EMT	Neutrophil Fibroblast	8, 17
			ICB resistance	Macrophage CD8^+^ T cell	18, 19
OIS	Oncogene activation	Anti-tumor	Recruitment of CD4^+^ T cells	Tumor cell EC	8, 20, 21
			Macrophage polarization towards M1	Fibroblast	22
		Pro-tumor	Tumorigenesis promotion	Macrophage Fibroblast	8, 23
			Metastasis promotion	Tumor cell EC	8, 13
			Invasion promotion	Tumor cell	8
			Chemoresistance	Tumor cell	8
			Immunosuppression	Fibroblast	8
SIPS	Stress signals	Pro-tumor	Tumorigenesis promotion(x2)	Fibroblast	24, 25
			Immunosuppression	Tumor cell	26
RS	Shortened telomere length	Pro-tumor	Angiogenesis	EC	27, 28
			Tumorigenesis promotion	Fibroblast	29
			Impaired immune surveillance	CD8^+^ T cell	30, 31
		Anti-tumor	Growth arrest	Tumor cell	32
Age-related immune dysfunction	Physiological aging	Pro-tumor	Macrophage polarization towards M2	Macrophage	33, 34
			Impaired immune surveillance	NK cell	35
			Metastasis promotion	Neutrophil	36
			Impaired antigen presentation	DC	37-39
			ICB adverse events	CD4^+^ T cell	40

TIS, therapy-induced senescence; OIS, oncogene-induced senescence; EC, endothelial cell; DC, dendritic cell; NK cell, natural killer cell; ICB, immune checkpoint blockade; PDAC, pancreatic ductal adenocarcinoma; EMT, epithelial-mesenchymal transition.

**Table 2:** Current clinical trials targeting senescence against cancers

Category	Drug	Mechanism	Combination therapy	Condition	Design	Reference	Status
Regulation of senescence	Dexamethasone	Induction of senescence	Anti-PD-1 therapy	NSCLC	Phase I/II	NCT04037462	Terminated
	Rapamycin	Inhibition of SASP	Alisertib	Advanced Solid Tumors	Phase I	292	With result
Clearance of senescence	D plus Q	1st-generation senolytics	None	Childhood Cancer	Phase II	NCT04733534	Recruiting
			Anti-PD-1 therapy	Head and Neck Cancer	Phase II	NCT05724329	Active
			None	Triple-negative Breast Cancer	Phase II	NCT06355037	Recruiting
	Fisetin	1st-generation senolytics	None	Childhood Cancer	Phase II	NCT04733534	Recruiting
			None	Breast Cancer	Phase II	NCT05595499	Recruiting
			None	Breast Cancer	Phase II	NCT06113016	Recruiting
	ABT-263 (Navitoclax)	1st-generation senolytics	Gemcitabine	Advanced solid tumors	Phase I	293	With result
			Docetaxel	Advanced solid tumors	Phase I	294	With result
			None	Lymphoid malignancies	Phase IIa	295	With result
			Rituximab	Chronic lymphocytic leukemia	Phase II	296	With result
	ABT-737	1st-generation senolytics	None	Ovarian Cancer	Observational study	NCT01440504	Completed
	“One-two” punch therapy	Induction of senescence plus senolytics	Decitabine plus navitoclax	Acute myeloid leukemia	Phase Ib	NCT05222984	Active
			Olaparib plus navitoclax	Triple-negative Breast Cancer	Phase I	NCT05358639	Active

NSCLC, non-small-cell lung cancer; D, dasatinib; Q, quercetin; SCLC, small-cell lung cancer.

**Table 3:** Preclinical studies clearing senescent immune and stromal cells within the tumor microenvironment.

Target	Treatment	Condition	Outcome	Reference
Neutrophil	3MRp16 model with ganciclovir suicide gene strategy	Prostate cancer	Suppressed tumor growth	107
	Procyanidin C1	Melanoma	Reduced tumor metastasis and restored T cell responses	128
Macrophage	Diphtheria toxin targeting tagged cells	Lung cancer	Diminished lung tumor burden and prolonged survival	23
	ABT-737			
	ABT-263	Lung cancer	Suppressed early tumorigenesis	138
	ABT-263	Colon cancer	Restored CD8^+^ T cell proliferation and response to immunotherapy	19
	Nicotinamide mononucleotide	Glioblastoma	Inhibited T-cell dysfunction and delayed tumor initiation	133
	IL-4	Aging	Improved the health span of aged mice	335
T cell	Metformin	NA	Lowered IFN-γ and IL-6 and increased TNF-α production	283
	CD153-CpG vaccine	Obesity	Improved obesity-induced metabolic disorders	333
Endothelial cell	Anti-Notch1/ VCAM1 antibody	Ovarian cancer	Reduced tumor cell adhesion and lowered lung metastasis	252
	GPNMB vaccine	Atherosclerosis	Improved metabolic disorders	334
Fibroblast	ABT-199	Pancreatic cancer	Restored CD8^+^ T cell function and response to immunotherapy	236
	ABT-737	Breast cancer	Enhanced NK cell function and infiltration	263
	Anti-TSPAN8 antibody	Breast cancer	Resensitize the response to chemotherapy	237
	Q	Osteosarcoma	Reduced tumor invasiveness	303
	D plus Q	Ovarian cancer	Reduced tumor metastasis	304

D, dasatinib; Q, quercetin; GPNMB, glycoprotein nonmetastatic melanoma protein B.

## Abbreviations

SASP: senescence-associated secretory phenotype
DDR: DNA damages response
HSCs: hepatic stellate cells
PGE2: prostaglandin E2
HCC: hepatocellular carcinoma
OIS: oncogene-induced senescence
TME: tumor microenvironment
SA-βGal: senescence-associated ß-galactosidase
ECM: extracellular matrix
BM: bone marrow
TREM2: triggering receptor expressed on myeloid cells 2
APOE: apolipoprotein E
ADCC: antibody-dependent cellular cytotoxicity
NETs: neutrophil extracellular traps
NO: nitric oxide
FTO: fat mass and obesity-associated protein
EMT: epithelial-mesenchymal transition
APCs: antigen-presenting cells
FasL: Fas ligand
MDSCs: myeloid-derived suppressor cells
IMCs: immature myeloid cells
MO-MDSCs: monocytic myeloid-derived suppressor cells
PMN-MDSCs: polymorphonuclear myeloid-derived suppressor cells
TRAIL: TNF-related apoptosis-inducing ligand
NKCC: NK cell cytotoxicity
cDCs: classical dendritic cells
pDCs: plasmacytoid dendritic cells
CTLs: cytotoxic T lymphocytes
NSCLC: non-small cell lung cancer
ACKR2: atypical chemokine receptor 2
KLRG1: killer cell lectin-like receptor subfamily G 1
ICB: immune checkpoint blockade
PFS: progression-free survival
ADCP: antibody-dependent cellular phagocytosis
CDC: complement-dependent cytotoxicity
TLSs: tertiary lymphoid structures
LTi cells: lymphoid tissue inducer cells
HEVs: high endothelial venules
PMBCs: peripheral blood mononuclear cells
CAFs: cancer-associated fibroblasts
iCAFs: inflammatory CAFs
myCAFs: myogenic CAFs
HDAC: histone deacetylase
PDAC: pancreatic ductal adenocarcinoma
ECs: endothelial cells
eNOS: endothelial nitric oxide synthase
CAR: engineered chimeric antigen receptor
SCAPs: senescent cells antiapoptotic pathways
D: dasatinib
Q: quercetin
UCB: umbilical cord blood
cAMP: cyclic adenosine monophosphate
ILT4: immunoglobulin-like transcript 4
TGF-β: transforming growth factor-β
HLA-G: human leukocyte antigen-G
PKA: protein kinase A
CREB: cAMP-response element binding protein
ATM: ataxia telangiectasia-mutated
ERK1/2: extracellular regulated protein kinases 1/2
STAT1/3: signal transducer and activator of transcription 1/3
CDK: cyclin-dependent kinases
Rb: retinoblastoma protein
TLR8: Toll-like receptors 8
GLUT: glucose transporter
MMP: matrix metalloproteinase
OPN: osteopontin
ECM: extracellular matrix
AREG: amphiregulin
ICAM-1: intercellular adhesion molecule-1
TNF-α: tumor necrosis factor-α
IL: interleukin
IFN-γ: interferon-γ
TGF-β: transforming growth factor-β
EVs: extracellular vesicles
VEGF: vascular endothelial growth factor
PD-1: programmed cell death protein 1
PD-L1: programmed cell death protein ligand 1
EMT: epithelial-mesenchymal transition
NK: natural killer
Th2: helper T cell 2
rDC: regulatory dendritic cell
IDO: indoleamine 2,3-dioxygenase
ABCB4: ATP-binding cassette subfamily B member 4
EphA2: erythropoietin-producing hepatocellular A2
ephrin-A1: recombinant human Ephrin A receptor 1
JAK: janus kinase
STAT: signal transducer and activator of transcription
NF-κB: nuclear factor-κB
cGAS: cyclic guanosine monophosphate-adenosine monophosphate synthase
STING: stimulator of interferon genes
mTOR: mammalian target of rapamycin
MAPKAPK2: mitogen-activated protein kinase-activated protein kinase 2
DDR: DNA damage response
BCL-2: B-cell lymphoma-2
PI3K: phosphatidylinositide 3-kinases
CAR-T: chimeric antigen receptor-T
GPNMB: glycoprotein nonmetastatic melanoma protein B
uPAR: urokinase-type plasminogen activator receptor
NKG2DLS: Natural killer group 2 member D ligands
T/P: trametinib and palbociclib

## References

[1] DePinho RA (2000). The age of cancer. Nature.

[2] López-Otín C, Pietrocola F, Roiz-Valle D, Galluzzi L, Kroemer G (2023). Meta-hallmarks of aging and cancer. Cell Metab.

[3] Hayflick L (1965). THE LIMITED IN VITRO LIFETIME OF HUMAN DIPLOID CELL STRAINS. Exp Cell Res.

[4] Akbar AN, Henson SM (2011). Are senescence and exhaustion intertwined or unrelated processes that compromise immunity?. Nat Rev Immunol.

[5] Hanahan D, Weinberg RA (2011). Hallmarks of cancer: the next generation. Cell.

[6] Hanahan D (2022). Hallmarks of Cancer: New Dimensions. Cancer Discov.

[7] Borrelli C, Ricci B, Vulpis E, Fionda C, Ricciardi MR, Petrucci MT (2018). Drug-Induced Senescent Multiple Myeloma Cells Elicit NK Cell Proliferation by Direct or Exosome-Mediated IL15 Trans-Presentation. Cancer Immunol Res.

[8] Faget DV, Ren QH, Stewart SA (2019). Unmasking senescence: context-dependent effects of SASP in cancer. Nat Rev Cancer.

[9] Marin I, Boix O, Garcia-Garijo A, Sirois I, Caballe A, Zarzuela E (2023). Cellular Senescence Is Immunogenic and Promotes Antitumor Immunity. Cancer Discov.

[10] Abu-Humaidan AH, Ismail MA, Ahmad FM, Al Shboul S, Barham R, Tadros JS (2024). Therapy-induced senescent cancer cells exhibit complement activation and increased complement regulatory protein expression. Immunol Cell Biol.

[11] Chibaya L, Murphy KC, DeMarco KD, Gopalan S, Liu H, Parikh CN (2023). EZH2 inhibition remodels the inflammatory senescence-associated secretory phenotype to potentiate pancreatic cancer immune surveillance. Nat Cancer.

[12] Hwang HJ, Lee YR, Kang D, Lee HC, Seo HR, Ryu JK (2020). Endothelial cells under therapy-induced senescence secrete CXCL11, which increases aggressiveness of breast cancer cells. Cancer Lett.

[13] Ma L, He X, Fu Y, Ge S, Yang Z (2024). Senescent endothelial cells promote liver metastasis of uveal melanoma in single-cell resolution. J Transl Med.

[14] Dai D, Pei Y, Zhu B, Wang D, Pei S, Huang H Chemoradiotherapy-induced ACKR2(+) tumor cells drive CD8(+) T cell senescence and cervical cancer recurrence. Cell Rep Med. 2024: 101550.

[15] Guan X, LaPak KM, Hennessey RC, Yu CY, Shakya R, Zhang J (2017). Stromal Senescence By Prolonged CDK4/6 Inhibition Potentiates Tumor Growth. Mol Cancer Res.

[16] Saleh T, Bloukh S, Hasan M, Al Shboul S (2023). Therapy-induced senescence as a component of tumor biology: Evidence from clinical cancer. Biochim Biophys Acta Rev Cancer.

[17] Ou B, Liu Y, Gao Z, Xu J, Yan Y, Li Y (2022). Senescent neutrophils-derived exosomal piRNA-17560 promotes chemoresistance and EMT of breast cancer via FTO-mediated m6A demethylation. Cell Death Dis.

[18] Ferrara R, Naigeon M, Auclin E, Duchemann B, Cassard L, Jouniaux JM (2021). Circulating T-cell Immunosenescence in Patients with Advanced Non-small Cell Lung Cancer Treated with Single-agent PD-1/PD-L1 Inhibitors or Platinum-based Chemotherapy. Clin Cancer Res.

[19] Maggiorani D, Le O, Lisi V, Landais S, Moquin-Beaudry G, Lavallée VP (2024). Senescence drives immunotherapy resistance by inducing an immunosuppressive tumor microenvironment. Nat Commun.

[20] Kang TW, Yevsa T, Woller N, Hoenicke L, Wuestefeld T, Dauch D (2011). Senescence surveillance of pre-malignant hepatocytes limits liver cancer development. Nature.

[21] Yin K, Patten D, Gough S, de Barros Gonçalves S, Chan A, Olan I (2022). Senescence-induced endothelial phenotypes underpin immune-mediated senescence surveillance. Genes Dev.

[22] Lujambio A, Akkari L, Simon J, Grace D, Tschaharganeh DF, Bolden JE (2013). Non-cell-autonomous tumor suppression by p53. Cell.

[23] Haston S, Gonzalez-Gualda E, Morsli S, Ge J, Reen V, Calderwood A (2023). Clearance of senescent macrophages ameliorates tumorigenesis in KRAS-driven lung cancer. Cancer Cell.

[24] Liu D, Hornsby PJ (2007). Senescent human fibroblasts increase the early growth of xenograft tumors via matrix metalloproteinase secretion. Cancer Res.

[25] Volonte D, Zou H, Bartholomew JN, Liu Z, Morel PA, Galbiati F (2015). Oxidative stress-induced inhibition of Sirt1 by caveolin-1 promotes p53-dependent premature senescence and stimulates the secretion of interleukin 6 (IL-6). J Biol Chem.

[26] Wang J, Tao Q, Pan Y, Wanyan Z, Zhu F, Xu X (2020). Stress-induced premature senescence activated by the SENEX gene mediates apoptosis resistance of diffuse large B-cell lymphoma via promoting immunosuppressive cells and cytokines. Immun Inflamm Dis.

[27] Coppé JP, Kauser K, Campisi J, Beauséjour CM (2006). Secretion of vascular endothelial growth factor by primary human fibroblasts at senescence. J Biol Chem.

[28] Yang F, Tuxhorn JA, Ressler SJ, McAlhany SJ, Dang TD, Rowley DR (2005). Stromal expression of connective tissue growth factor promotes angiogenesis and prostate cancer tumorigenesis. Cancer Res.

[29] Krtolica A, Parrinello S, Lockett S, Desprez PY, Campisi J (2001). Senescent fibroblasts promote epithelial cell growth and tumorigenesis: a link between cancer and aging. Proc Natl Acad Sci U S A.

[30] Appay V, Nixon DF, Donahoe SM, Gillespie GM, Dong T, King A (2000). HIV-specific CD8(+) T cells produce antiviral cytokines but are impaired in cytolytic function. J Exp Med.

[31] Yang OO, Lin H, Dagarag M, Ng HL, Effros RB, Uittenbogaart CH (2005). Decreased perforin and granzyme B expression in senescent HIV-1-specific cytotoxic T lymphocytes. Virology.

[32] Rodriguez-Brenes IA, Wodarz D, Komarova NL (2015). Quantifying replicative senescence as a tumor suppressor pathway and a target for cancer therapy. Sci Rep.

[33] Nyugen J, Agrawal S, Gollapudi S, Gupta S (2010). Impaired functions of peripheral blood monocyte subpopulations in aged humans. J Clin Immunol.

[34] Wang Y, Wehling-Henricks M, Samengo G, Tidball JG (2015). Increases of M2a macrophages and fibrosis in aging muscle are influenced by bone marrow aging and negatively regulated by muscle-derived nitric oxide. Aging Cell.

[35] Sanchez-Correa B, Morgado S, Gayoso I, Bergua JM, Casado JG, Arcos MJ (2011). Human NK cells in acute myeloid leukaemia patients: analysis of NK cell-activating receptors and their ligands. Cancer Immunol Immunother.

[36] Yang C, Wang Z, Li L, Zhang Z, Jin X, Wu P (2021). Aged neutrophils form mitochondria-dependent vital NETs to promote breast cancer lung metastasis. J Immunother Cancer.

[37] Agrawal A, Agrawal S, Cao JN, Su H, Osann K, Gupta S (2007). Altered innate immune functioning of dendritic cells in elderly humans: a role of phosphoinositide 3-kinase-signaling pathway. J Immunol.

[38] Grolleau-Julius A, Harning EK, Abernathy LM, Yung RL (2008). Impaired dendritic cell function in aging leads to defective antitumor immunity. Cancer Res.

[39] Pereira LF, de Souza AP, Borges TJ, Bonorino C (2011). Impaired in vivo CD4+ T cell expansion and differentiation in aged mice is not solely due to T cell defects: decreased stimulation by aged dendritic cells. Mech Ageing Dev.

[40] Tsukamoto H, Komohara Y, Tomita Y, Miura Y, Motoshima T, Imamura K (2022). Aging-associated and CD4 T-cell-dependent ectopic CXCL13 activation predisposes to anti-PD-1 therapy-induced adverse events. Proc Natl Acad Sci U S A.

[41] Birch J, Gil J (2020). Senescence and the SASP: many therapeutic avenues. Genes Dev.

[42] Hanna A, Balko JM (2023). No rest for the wicked: Tumor cell senescence reshapes the immune microenvironment. Cancer Cell.

[43] Prasanna PG, Citrin DE, Hildesheim J, Ahmed MM, Venkatachalam S, Riscuta G (2021). Therapy-Induced Senescence: Opportunities to Improve Anticancer Therapy. J Natl Cancer Inst.

[44] Schmitt CA, Wang B, Demaria M (2022). Senescence and cancer - role and therapeutic opportunities. Nat Rev Clin Oncol.

[45] Wang L, Lankhorst L, Bernards R (2022). Exploiting senescence for the treatment of cancer. Nat Rev Cancer.

[46] Bloom SI, Islam MT, Lesniewski LA, Donato AJ (2023). Mechanisms and consequences of endothelial cell senescence. Nat Rev Cardiol.

[47] Gabai Y, Assouline B, Ben-Porath I (2023). Senescent stromal cells: roles in the tumor microenvironment. Trends Cancer.

[48] Lian JY, Yue Y, Yu WN, Zhang Y (2020). Immunosenescence: a key player in cancer development. J Hematol Oncol.

[49] Liu Z, Liang Q, Ren Y, Guo C, Ge X, Wang L (2023). Immunosenescence: molecular mechanisms and diseases. Signal Transduct Target Ther.

[50] Solana R, Tarazona R, Gayoso I, Lesur O, Dupuis G, Fulop T (2012). Innate immunosenescence: Effect of aging on cells and receptors of the innate immune system in humans. Seminars in Immunology.

[51] López-Otín C, Blasco MA, Partridge L, Serrano M, Kroemer G (2023). Hallmarks of aging: An expanding universe. Cell.

[52] Gorgoulis V, Adams PD, Alimonti A, Bennett DC, Bischof O, Bishop C (2019). Cellular Senescence: Defining a Path Forward. Cell.

[53] Campisi J, di Fagagna FD (2007). Cellular senescence: when bad things happen to good cells. Nat Rev Mol Cell Bio.

[54] Coppé JP, Desprez PY, Krtolica A, Campisi J (2010). The Senescence-Associated Secretory Phenotype: The Dark Side of Tumor Suppression. Annu Rev Pathol-Mech.

[55] Kim Y, Jang Y, Kim MS, Kang C (2024). Metabolic remodeling in cancer and senescence and its therapeutic implications. Trends Endocrinol Metab.

[56] Zhang Y, Tang J, Jiang C, Yi H, Guang S, Yin G (2025). Metabolic reprogramming in cancer and senescence. MedComm (2020).

[57] Favaro E, Bensaad K, Chong MG, Tennant DA, Ferguson DJ, Snell C (2012). Glucose utilization via glycogen phosphorylase sustains proliferation and prevents premature senescence in cancer cells. Cell Metab.

[58] Li F, Liu P, Mi W, Li L, Anderson NM, Lesner NP (2024). Blocking methionine catabolism induces senescence and confers vulnerability to GSK3 inhibition in liver cancer. Nat Cancer.

[59] Wang Z, Gao J, Xu C (2024). Targeting metabolism to influence cellular senescence a promising anti-cancer therapeutic strategy. Biomed Pharmacother.

[60] de Magalhães JP, Passos JF (2018). Stress, cell senescence and organismal ageing. Mech Ageing Dev.

[61] Hornsby PJ (2007). Senescence as an anticancer mechanism. J Clin Oncol.

[62] Mikuła-Pietrasik J, Niklas A, Uruski P, Tykarski A, Książek K (2020). Mechanisms and significance of therapy-induced and spontaneous senescence of cancer cells. Cell Mol Life Sci.

[63] Elshazly AM, Shahin U, Al Shboul S, Gewirtz DA, Saleh T (2024). A Conversation with ChatGPT on Contentious Issues in Senescence and Cancer Research. Mol Pharmacol.

[64] Liu XL, Ding J, Meng LH (2018). Oncogene-induced senescence: a double edged sword in cancer. Acta Pharmacol Sin.

[65] Almanzar G, Schwaiger S, Jenewein B, Keller M, Herndler-Brandstetter D, Würzner R (2005). Long-term cytomegalovirus infection leads to significant changes in the composition of the CD8 T-cell repertoire, which may be the basis for an imbalance in the cytokine production profile in elderly persons. J Virol.

[66] Kuilman T, Michaloglou C, Vredeveld LCW, Douma S, van Doom R, Desmet CJ (2008). Oncogene-induced senescence relayed by an interleukin-dependent inflammatory network. Cell.

[67] Nikolich-Zugich J (2008). Ageing and life-long maintenance of T-cell subsets in the face of latent persistent infections. Nat Rev Immunol.

[68] Suen H, Brown R, Yang S, Weatherburn C, Ho PJ, Woodland N (2016). Multiple myeloma causes clonal T-cell immunosenescence: identification of potential novel targets for promoting tumour immunity and implications for checkpoint blockade. Leukemia.

[69] Lynch HE, Goldberg GL, Chidgey A, Van den Brink MR, Boyd R, Sempowski GD (2009). Thymic involution and immune reconstitution. Trends Immunol.

[70] Rezzani R, Nardo L, Favero G, Peroni M, Rodella LF (2014). Thymus and aging: morphological, radiological, and functional overview. Age (Dordr).

[71] Hohensinner PJ, Goronzy JJ, Weyand CM (2011). Telomere dysfunction, autoimmunity and aging. Aging Dis.

[72] Ferrando-Martínez S, Romero-Sánchez MC, Solana R, Delgado J, de la Rosa R, Muñoz-Fernández MA (2013). Thymic function failure and C-reactive protein levels are independent predictors of all-cause mortality in healthy elderly humans. Age.

[73] Franceschi C, Garagnani P, Parini P, Giuliani C, Santoro A (2018). Inflammaging: a new immune-metabolic viewpoint for age-related diseases. Nat Rev Endocrinol.

[74] Franceschi C, Bonafè M, Valensin S, Olivieri F, De Luca M, Ottaviani E (2000). Inflamm-aging. An evolutionary perspective on immunosenescence. Ann N Y Acad Sci.

[75] Fulop T, Larbi A, Dupuis G, Le Page A, Frost EH, Cohen AA (2017). Immunosenescence and Inflamm-Aging As Two Sides of the Same Coin: Friends or Foes?. Front Immunol.

[76] Santoro A, Bientinesi E, Monti D (2021). Immunosenescence and inflammaging in the aging process: age-related diseases or longevity?. Ageing Res Rev.

[77] Yousefzadeh MJ, Flores RR, Zhu Y, Schmiechen ZC, Brooks RW, Trussoni CE (2021). An aged immune system drives senescence and ageing of solid organs. Nature.

[78] Montégut L, López-Otín C, Kroemer G (2024). Aging and cancer. Mol Cancer.

[79] de Magalhães JP (2024). Cellular senescence in normal physiology. Science.

[80] Burgess DJ (2011). Senescence: Tumorigenesis under surveillance. Nat Rev Cancer.

[81] Cai X, Yin G, Chen S, Tacke F, Guillot A, Liu H (2024). CDK4/6 inhibition enhances T-cell immunotherapy on hepatocellular carcinoma cells by rejuvenating immunogenicity. Cancer Cell Int.

[82] Ohtani N, Takahashi A, Mann DJ, Hara E (2012). Cellular senescence: a double-edged sword in the fight against cancer. Exp Dermatol.

[83] Ewald JA, Desotelle JA, Wilding G, Jarrard DF (2010). Therapy-Induced Senescence in Cancer. Jnci-J Natl Cancer I.

[84] Onyema OO, Decoster L, Njemini R, Forti LN, Bautmans I, De Waele M (2015). Chemotherapy-induced Changes and Immunosenescence of CD8 T-Cells in Patients with Breast Cancer. Anticancer Res.

[85] Tabasso AFS, Jones DJL, Jones GDD, Macip S (2019). Radiotherapy-Induced Senescence and its Effects on Responses to Treatment. Clin Oncol-Uk.

[86] Takasugi M, Yoshida Y, Ohtani N (2022). Cellular senescence and the tumour microenvironment. Mol Oncol.

[87] Amor C, Feucht J, Leibold J, Ho YJ, Zhu C, Alonso-Curbelo D (2020). Senolytic CAR T cells reverse senescence-associated pathologies. Nature.

[88] Amor C, Feucht J, Leibold J, Ho YJ, Zhu C, Alonso-Curbelo D (2024). Author Correction: Senolytic CAR T cells reverse senescence-associated pathologies. Nature.

[89] Ruscetti M, Morris JPt, Mezzadra R, Russell J, Leibold J, Romesser PB (2020). Senescence-Induced Vascular Remodeling Creates Therapeutic Vulnerabilities in Pancreas Cancer. Cell.

[90] Ruscetti M, Morris JPt, Mezzadra R, Russell J, Leibold J, Romesser PB (2021). Senescence-Induced Vascular Remodeling Creates Therapeutic Vulnerabilities in Pancreas Cancer. Cell.

[91] Wang B, Kohli J, Demaria M (2020). Senescent Cells in Cancer Therapy: Friends or Foes?. Trends Cancer.

[92] Chien Y, Scuoppo C, Wang X, Fang X, Balgley B, Bolden JE (2011). Control of the senescence-associated secretory phenotype by NF-κB promotes senescence and enhances chemosensitivity. Genes Dev.

[93] Liu Y, Hawkins OE, Su Y, Vilgelm AE, Sobolik T, Thu YM (2013). Targeting aurora kinases limits tumour growth through DNA damage-mediated senescence and blockade of NF-κB impairs this drug-induced senescence. EMBO Mol Med.

[94] Evangelou K, Belogiannis K, Papaspyropoulos A, Petty R, Gorgoulis VG (2023). Escape from senescence: molecular basis and therapeutic ramifications. J Pathol.

[95] Domen A, Deben C, De Pauw I, Hermans C, Lambrechts H, Verswyvel J (2022). Prognostic implications of cellular senescence in resected non-small cell lung cancer. Transl Lung Cancer Res.

[96] Wei Q, Chen R, He X, Qu Y, Yan C, Liu X (2024). Multi-omics and single cell characterization of cancer immunosenescence landscape. Sci Data.

[97] Dou X, Fu Q, Long Q, Liu S, Zou Y, Fu D (2023). PDK4-dependent hypercatabolism and lactate production of senescent cells promotes cancer malignancy. Nat Metab.

[98] Fu D, Zhang B, Fan W, Zeng F, Feng J, Wang X (2024). Fatty acid metabolism prognostic signature predicts tumor immune microenvironment and immunotherapy, and identifies tumorigenic role of MOGAT2 in lung adenocarcinoma. Front Immunol.

[99] Li Q, Liu H (2025). Identification of Prognostic Genes Related to Cell Senescence and Lipid Metabolism in Glioblastoma Based on Transcriptome and Single-Cell RNA-Seq Data. Int J Mol Sci.

[100] Pacifico F, Mellone S, D'Incalci M, Stornaiuolo M, Leonardi A, Crescenzi E (2022). Trabectedin suppresses escape from therapy-induced senescence in tumor cells by interfering with glutamine metabolism. Biochem Pharmacol.

[101] Chen L, Zhang W, Chen D, Yang Q, Sun S, Dai Z (2023). RBM4 dictates ESCC cell fate switch from cellular senescence to glutamine-addiction survival through inhibiting LKB1-AMPK-axis. Signal Transduct Target Ther.

[102] Lai P, Liu L, Bancaro N, Troiani M, Calì B, Li Y (2025). Mitochondrial DNA released by senescent tumor cells enhances PMN-MDSC-driven immunosuppression through the cGAS-STING pathway. Immunity.

[103] Cao LL, Kagan JC (2023). Targeting innate immune pathways for cancer immunotherapy. Immunity.

[104] Galassi C, Chan TA, Vitale I, Galluzzi L (2024). The hallmarks of cancer immune evasion. Cancer Cell.

[105] Liew PX, Kubes P (2019). The Neutrophil's Role During Health and Disease. Physiol Rev.

[106] Tortorella C, Simone O, Piazzolla G, Stella I, Cappiello V, Antonaci S (2006). Role of phosphoinositide 3-kinase and extracellular signal-regulated kinase pathways in granulocyte macrophage-colony-stimulating factor failure to delay fas-induced neutrophil apoptosis in elderly humans. J Gerontol A Biol Sci Med Sci.

[107] Bancaro N, Calì B, Troiani M, Elia AR, Arzola RA, Attanasio G (2023). Apolipoprotein E induces pathogenic senescent-like myeloid cells in prostate cancer. Cancer Cell.

[108] Chatta GS, Andrews RG, Rodger E, Schrag M, Hammond WP, Dale DC (1993). Hematopoietic progenitors and aging: alterations in granulocytic precursors and responsiveness to recombinant human G-CSF, GM-CSF, and IL-3. J Gerontol.

[109] Chatta GS, Price TH, Stratton JR, Dale DC (1994). Aging and marrow neutrophil reserves. J Am Geriatr Soc.

[110] Simell B, Vuorela A, Ekström N, Palmu A, Reunanen A, Meri S (2011). Aging reduces the functionality of anti-pneumococcal antibodies and the killing of Streptococcus pneumoniae by neutrophil phagocytosis. Vaccine.

[111] Simmons SR, Tchalla EYI, Bhalla M, Bou Ghanem EN (2022). The Age-Driven Decline in Neutrophil Function Contributes to the Reduced Efficacy of the Pneumococcal Conjugate Vaccine in Old Hosts. Front Cell Infect Microbiol.

[112] Liu Y, Cao X (2016). Characteristics and Significance of the Pre-metastatic Niche. Cancer Cell.

[113] Peng Z, Liu C, Victor AR, Cao DY, Veiras LC, Bernstein EA (2021). Tumors exploit CXCR4(hi)CD62L(lo) aged neutrophils to facilitate metastatic spread. Oncoimmunology.

[114] Que H, Fu Q, Lan T, Tian X, Wei X (2022). Tumor-associated neutrophils and neutrophil-targeted cancer therapies. Biochim Biophys Acta Rev Cancer.

[115] Behrens LM, van Egmond M, van den Berg TK (2023). Neutrophils as immune effector cells in antibody therapy in cancer. Immunol Rev.

[116] Fülöp T Jr, Fóris G, Wórum I, Leövey A (1985). Age-dependent alterations of Fc gamma receptor-mediated effector functions of human polymorphonuclear leucocytes. Clin Exp Immunol.

[117] Guettinger Y, Barbin K, Peipp M, Bruenke J, Dechant M, Horner H (2010). A recombinant bispecific single-chain fragment variable specific for HLA class II and Fc alpha RI (CD89) recruits polymorphonuclear neutrophils for efficient lysis of malignant B lymphoid cells. J Immunol.

[118] Stockmeyer B, Dechant M, van Egmond M, Tutt AL, Sundarapandiyan K, Graziano RF (2000). Triggering Fc alpha-receptor I (CD89) recruits neutrophils as effector cells for CD20-directed antibody therapy. J Immunol.

[119] Eruslanov EB, Bhojnagarwala PS, Quatromoni JG, Stephen TL, Ranganathan A, Deshpande C (2014). Tumor-associated neutrophils stimulate T cell responses in early-stage human lung cancer. J Clin Invest.

[120] Singhal S, Bhojnagarwala PS, O'Brien S, Moon EK, Garfall AL, Rao AS (2016). Origin and Role of a Subset of Tumor-Associated Neutrophils with Antigen-Presenting Cell Features in Early-Stage Human Lung Cancer. Cancer Cell.

[121] Wu Y, Ma J, Yang X, Nan F, Zhang T, Ji S (2024). Neutrophil profiling illuminates anti-tumor antigen-presenting potency. Cell.

[122] Gershkovitz M, Caspi Y, Fainsod-Levi T, Katz B, Michaeli J, Khawaled S (2018). TRPM2 Mediates Neutrophil Killing of Disseminated Tumor Cells. Cancer Res.

[123] Erdman SE, Rao VP, Poutahidis T, Rogers AB, Taylor CL, Jackson EA (2009). Nitric oxide and TNF-alpha trigger colonic inflammation and carcinogenesis in Helicobacter hepaticus-infected, Rag2-deficient mice. Proc Natl Acad Sci U S A.

[124] Schmielau J, Finn OJ (2001). Activated granulocytes and granulocyte-derived hydrogen peroxide are the underlying mechanism of suppression of t-cell function in advanced cancer patients. Cancer Res.

[125] Ogawa K, Suzuki K, Okutsu M, Yamazaki K, Shinkai S (2008). The association of elevated reactive oxygen species levels from neutrophils with low-grade inflammation in the elderly. Immun Ageing.

[126] Adrover JM, McDowell SAC, He XY, Quail DF, Egeblad M (2023). NETworking with cancer: The bidirectional interplay between cancer and neutrophil extracellular traps. Cancer Cell.

[127] Hazeldine J, Harris P, Chapple IL, Grant M, Greenwood H, Livesey A (2014). Impaired neutrophil extracellular trap formation: a novel defect in the innate immune system of aged individuals. Aging Cell.

[128] Duan R, Jiang L, Wang T, Li Z, Yu X, Gao Y (2024). Aging-induced immune microenvironment remodeling fosters melanoma in male mice via γδ17-Neutrophil-CD8 axis. Nat Commun.

[129] Kalafati L, Kourtzelis I, Schulte-Schrepping J, Li X, Hatzioannou A, Grinenko T (2020). Innate Immune Training of Granulopoiesis Promotes Anti-tumor Activity. Cell.

[130] Xiang X, Wang J, Lu D, Xu X (2021). Targeting tumor-associated macrophages to synergize tumor immunotherapy. Signal Transduct Target Ther.

[131] Narasimhan PB, Marcovecchio P, Hamers AAJ, Hedrick CC (2019). Nonclassical Monocytes in Health and Disease. Annu Rev Immunol.

[132] Gu M, Liu Y, Zheng W, Jing Z, Li X, Guo W (2024). Combined targeting of senescent cells and senescent macrophages: A new idea for integrated treatment of lung cancer. Semin Cancer Biol.

[133] Wada H, Otsuka R, Germeraad WTV, Murata T, Kondo T, Seino KI (2023). Tumor cell-induced macrophage senescence plays a pivotal role in tumor initiation followed by stable growth in immunocompetent condition. J Immunother Cancer.

[134] Cassetta L, Pollard JW (2023). A timeline of tumour-associated macrophage biology. Nat Rev Cancer.

[135] Angarola BL, Sharma S, Katiyar N, Kang HG, Nehar-Belaid D, Park S (2025). Comprehensive single-cell aging atlas of healthy mammary tissues reveals shared epigenomic and transcriptomic signatures of aging and cancer. Nat Aging.

[136] van Duin D, Allore HG, Mohanty S, Ginter S, Newman FK, Belshe RB (2007). Prevaccine determination of the expression of costimulatory B7 molecules in activated monocytes predicts influenza vaccine responses in young and older adults. J Infect Dis.

[137] Villanueva JL, Solana R, Alonso MC, Peña J (1990). Changes in the expression of HLA-class II antigens on peripheral blood monocytes from aged humans. Dis Markers.

[138] Prieto LI, Sturmlechner I, Graves SI, Zhang C, Goplen NP, Yi ES (2023). Senescent alveolar macrophages promote early-stage lung tumorigenesis. Cancer Cell.

[139] Chiu BC, Stolberg VR, Chensue SW (2008). Mononuclear phagocyte-derived IL-10 suppresses the innate IL-12/IFN-gamma axis in lung-challenged aged mice. J Immunol.

[140] Kelly J, Ali Khan A, Yin J, Ferguson TA, Apte RS (2007). Senescence regulates macrophage activation and angiogenic fate at sites of tissue injury in mice. J Clin Invest.

[141] Gabrilovich DI, Nagaraj S (2009). Myeloid-derived suppressor cells as regulators of the immune system. Nat Rev Immunol.

[142] Jackaman C, Tomay F, Duong L, Abdol Razak NB, Pixley FJ, Metharom P (2017). Aging and cancer: The role of macrophages and neutrophils. Ageing Res Rev.

[143] Ruhland MK, Loza AJ, Capietto AH, Luo X, Knolhoff BL, Flanagan KC (2016). Stromal senescence establishes an immunosuppressive microenvironment that drives tumorigenesis. Nat Commun.

[144] Enioutina EY, Bareyan D, Daynes RA (2011). A role for immature myeloid cells in immune senescence. J Immunol.

[145] Flores RR, Clauson CL, Cho J, Lee BC, McGowan SJ, Baker DJ (2017). Expansion of myeloid-derived suppressor cells with aging in the bone marrow of mice through a NF-κB-dependent mechanism. Aging Cell.

[146] Chandra D, Jahangir A, Quispe-Tintaya W, Einstein MH, Gravekamp C (2013). Myeloid-derived suppressor cells have a central role in attenuated Listeria monocytogenes-based immunotherapy against metastatic breast cancer in young and old mice. Br J Cancer.

[147] Okuma A, Hanyu A, Watanabe S, Hara E (2017). p16(Ink4a) and p21(Cip1/Waf1) promote tumour growth by enhancing myeloid-derived suppressor cells chemotaxis. Nat Commun.

[148] Zhou Y, Cheng L, Liu L, Li X (2023). NK cells are never alone: crosstalk and communication in tumour microenvironments. Mol Cancer.

[149] Bruno A, Ferlazzo G, Albini A, Noonan DM (2014). A think tank of TINK/TANKs: tumor-infiltrating/tumor-associated natural killer cells in tumor progression and angiogenesis. J Natl Cancer Inst.

[150] Brighton PJ, Maruyama Y, Fishwick K, Vrljicak P, Tewary S, Fujihara R (2017). Clearance of senescent decidual cells by uterine natural killer cells in cycling human endometrium. Elife.

[151] Brauning A, Rae M, Zhu G, Fulton E, Admasu TD, Stolzing A (2022). Aging of the Immune System: Focus on Natural Killer Cells Phenotype and Functions. Cells.

[152] Borrego F, Alonso MC, Galiani MD, Carracedo J, Ramirez R, Ostos B (1999). NK phenotypic markers and IL2 response in NK cells from elderly people. Exp Gerontol.

[153] Hazeldine J, Hampson P, Lord JM (2012). Reduced release and binding of perforin at the immunological synapse underlies the age-related decline in natural killer cell cytotoxicity. Aging Cell.

[154] Le Garff-Tavernier M, Béziat V, Decocq J, Siguret V, Gandjbakhch F, Pautas E (2010). Human NK cells display major phenotypic and functional changes over the life span. Aging Cell.

[155] Krishnaraj R, Bhooma T (1996). Cytokine sensitivity of human NK cells during immunosenescence. 2. IL2-induced interferon gamma secretion. Immunol Lett.

[156] Mariani E, Pulsatelli L, Meneghetti A, Dolzani P, Mazzetti I, Neri S (2001). Different IL-8 production by T and NK lymphocytes in elderly subjects. Mech Ageing Dev.

[157] Wculek SK, Cueto FJ, Mujal AM, Melero I, Krummel MF, Sancho D (2020). Dendritic cells in cancer immunology and immunotherapy. Nat Rev Immunol.

[158] Sridharan A, Esposo M, Kaushal K, Tay J, Osann K, Agrawal S (2011). Age-associated impaired plasmacytoid dendritic cell functions lead to decreased CD4 and CD8 T cell immunity. Age (Dordr).

[159] Guo Z, Tilburgs T, Wong B, Strominger JL (2014). Dysfunction of dendritic cells in aged C57BL/6 mice leads to failure of natural killer cell activation and of tumor eradication. Proc Natl Acad Sci U S A.

[160] Speiser DE, Chijioke O, Schaeuble K, Münz C (2023). CD4(+) T cells in cancer. Nat Cancer.

[161] Han S, Georgiev P, Ringel AE, Sharpe AH, Haigis MC (2023). Age-associated remodeling of T cell immunity and metabolism. Cell Metab.

[162] Voehringer D, Blaser C, Brawand P, Raulet DH, Hanke T, Pircher H (2001). Viral infections induce abundant numbers of senescent CD8 T cells. J Immunol.

[163] Saavedra D, García B, Lorenzo-Luaces P, González A, Popa X, Fuentes KP (2016). Biomarkers related to immunosenescence: relationships with therapy and survival in lung cancer patients. Cancer Immunol Immunother.

[164] Ma M, Yang Y, Chen Z, Li X, Yang Z, Wang K (2023). T-cell senescence induced by peripheral phospholipids. Cell Biol Toxicol.

[165] Gao A, Liu X, Lin W, Wang J, Wang S, Si F (2021). Tumor-derived ILT4 induces T cell senescence and suppresses tumor immunity. J Immunother Cancer.

[166] Ye J, Ma C, Hsueh EC, Dou J, Mo W, Liu S (2014). TLR8 signaling enhances tumor immunity by preventing tumor-induced T-cell senescence. EMBO Mol Med.

[167] Ye J, Peng G (2015). Controlling T cell senescence in the tumor microenvironment for tumor immunotherapy. Oncoimmunology.

[168] Ma F, Liu X, Zhang Y, Tao Y, Zhao L, Abusalamah H (2025). Tumor extracellular vesicle-derived PD-L1 promotes T cell senescence through lipid metabolism reprogramming. Sci Transl Med.

[169] Ikeda H, Kawase K, Nishi T, Watanabe T, Takenaga K, Inozume T (2025). Immune evasion through mitochondrial transfer in the tumour microenvironment. Nature.

[170] Bandrés E, Merino J, Vázquez B, Inogés S, Moreno C, Subirá ML (2000). The increase of IFN-gamma production through aging correlates with the expanded CD8(+high)CD28(-)CD57(+) subpopulation. Clin Immunol.

[171] Brenchley JM, Karandikar NJ, Betts MR, Ambrozak DR, Hill BJ, Crotty LE (2003). Expression of CD57 defines replicative senescence and antigen-induced apoptotic death of CD8 T cells. Blood.

[172] Montes CL, Chapoval AI, Nelson J, Orhue V, Zhang X, Schulze DH (2008). Tumor-induced senescent T cells with suppressor function: A potential form of tumor immune evasion. Cancer Res.

[173] Huff WX, Bam M, Shireman JM, Kwon JH, Song L, Newman S (2021). Aging- and Tumor-Mediated Increase in CD8(+)CD28(-) T Cells Might Impose a Strong Barrier to Success of Immunotherapy in Glioblastoma. Immunohorizons.

[174] Onyema OO, Decoster L, Njemini R, Forti LN, Bautmans I, De Waele M (2015). Shifts in subsets of CD8+T-cells as evidence of immunosenescence in patients with cancers affecting the lungs: an observational case-control study. Bmc Cancer.

[175] Tsukishiro T, Donnenberg AD, Whiteside TL (2003). Rapid turnover of the CD8CD28 T-cell subset of effector cells in the circulation of patients with head and neck cancer. Cancer Immunol Immun.

[176] Zhang SP, Ke X, Zeng SY, Wu M, Lou JF, Wu L (2015). Analysis of CD8 Treg cells in patients with ovarian cancer: a possible mechanism for immune impairment. Cell Mol Immunol.

[177] Crespo J, Sun H, Welling TH, Tian Z, Zou W (2013). T cell anergy, exhaustion, senescence, and stemness in the tumor microenvironment. Curr Opin Immunol.

[178] Henson SM, Lanna A, Riddell NE, Franzese O, Macaulay R, Griffiths SJ (2014). p38 signaling inhibits mTORC1-independent autophagy in senescent human CD8⁺ T cells. J Clin Invest.

[179] Knaus HA, Berglund S, Hackl H, Blackford AL, Zeidner JF, Montiel-Esparza R (2018). Signatures of CD8+ T cell dysfunction in AML patients and their reversibility with response to chemotherapy. JCI Insight.

[180] Shirakawa K, Yan X, Shinmura K, Endo J, Kataoka M, Katsumata Y (2016). Obesity accelerates T cell senescence in murine visceral adipose tissue. J Clin Invest.

[181] Ikeda H, Kawase K, Nishi T, Watanabe T, Takenaga K, Inozume T (2025). Immune evasion through mitochondrial transfer in the tumour microenvironment. Nature.

[182] Libri V, Azevedo RI, Jackson SE, Di Mitri D, Lachmann R, Fuhrmann S (2011). Cytomegalovirus infection induces the accumulation of short-lived, multifunctional CD4+CD45RA+CD27+ T cells: the potential involvement of interleukin-7 in this process. Immunology.

[183] Callender LA, Carroll EC, Beal RWJ, Chambers ES, Nourshargh S, Akbar AN (2018). Human CD8(+) EMRA T cells display a senescence-associated secretory phenotype regulated by p38 MAPK. Aging Cell.

[184] Ramello MC, Tosello Boari J, Canale FP, Mena HA, Negrotto S, Gastman B (2014). Tumor-induced senescent T cells promote the secretion of pro-inflammatory cytokines and angiogenic factors by human monocytes/macrophages through a mechanism that involves Tim-3 and CD40L. Cell Death Dis.

[185] Liu L, Hao Z, Yang X, Li Y, Wang S, Li L (2025). Metabolic reprogramming in T cell senescence: a novel strategy for cancer immunotherapy. Cell Death Discov.

[186] Møller SH, Hsueh PC, Yu YR, Zhang L, Ho PC (2022). Metabolic programs tailor T cell immunity in viral infection, cancer, and aging. Cell Metab.

[187] Liu X, Hartman CL, Li L, Albert CJ, Si F, Gao A (2021). Reprogramming lipid metabolism prevents effector T cell senescence and enhances tumor immunotherapy. Sci Transl Med.

[188] McLane LM, Abdel-Hakeem MS, Wherry EJ (2019). CD8 T Cell Exhaustion During Chronic Viral Infection and Cancer. Annu Rev Immunol.

[189] Tedeschi V, Paldino G, Kunkl M, Paroli M, Sorrentino R, Tuosto L (2022). CD8(+) T Cell Senescence: Lights and Shadows in Viral Infections, Autoimmune Disorders and Cancer. Int J Mol Sci.

[190] Hui E, Cheung J, Zhu J, Su X, Taylor MJ, Wallweber HA (2017). T cell costimulatory receptor CD28 is a primary target for PD-1-mediated inhibition. Science.

[191] Kamphorst AO, Wieland A, Nasti T, Yang S, Zhang R, Barber DL (2017). Rescue of exhausted CD8 T cells by PD-1-targeted therapies is CD28-dependent. Science.

[192] Hudson WH, Gensheimer J, Hashimoto M, Wieland A, Valanparambil RM, Li P (2019). Proliferating Transitory T Cells with an Effector-like Transcriptional Signature Emerge from PD-1(+) Stem-like CD8(+) T Cells during Chronic Infection. Immunity.

[193] Fornara O, Odeberg J, Wolmer Solberg N, Tammik C, Skarman P, Peredo I (2015). Poor survival in glioblastoma patients is associated with early signs of immunosenescence in the CD4 T-cell compartment after surgery. Oncoimmunology.

[194] Gonnin C, Leemans M, Canoui-Poitrine F, Lebraud M, Corneau A, Roquebert L (2024). CD57(+) EMRA CD8(+) T cells in cancer patients over 70: associations with prior chemotherapy and response to anti-PD-1/PD-L1 therapy. Immun Ageing.

[195] Kim KH, Pyo H, Lee H, Oh D, Noh JM, Ahn YC (2023). Association of T Cell Senescence with Radiation Pneumonitis in Patients with Non-small Cell Lung Cancer. Int J Radiat Oncol Biol Phys.

[196] Song G, Wang X, Jia J, Yuan Y, Wan F, Zhou X (2013). Elevated level of peripheral CD8(+)CD28(-) T lymphocytes are an independent predictor of progression-free survival in patients with metastatic breast cancer during the course of chemotherapy. Cancer Immunol Immunother.

[197] Kugel CH 3rd, Douglass SM, Webster MR, Kaur A, Liu Q, Yin X (2018). Age Correlates with Response to Anti-PD1, Reflecting Age-Related Differences in Intratumoral Effector and Regulatory T-Cell Populations. Clin Cancer Res.

[198] Wang TW, Johmura Y, Suzuki N, Omori S, Migita T, Yamaguchi K (2022). Blocking PD-L1-PD-1 improves senescence surveillance and ageing phenotypes. Nature.

[199] Li C, Jiang P, Wei S, Xu X, Wang J (2020). Regulatory T cells in tumor microenvironment: new mechanisms, potential therapeutic strategies and future prospects. Mol Cancer.

[200] Liu X, Mo W, Ye J, Li L, Zhang Y, Hsueh EC (2018). Regulatory T cells trigger effector T cell DNA damage and senescence caused by metabolic competition. Nat Commun.

[201] Ye J, Huang X, Hsueh EC, Zhang Q, Ma C, Zhang Y (2012). Human regulatory T cells induce T-lymphocyte senescence. Blood.

[202] Ye J, Ma C, Hsueh EC, Eickhoff CS, Zhang Y, Varvares MA (2013). Tumor-derived γδ regulatory T cells suppress innate and adaptive immunity through the induction of immunosenescence. J Immunol.

[203] Garg SK, Delaney C, Toubai T, Ghosh A, Reddy P, Banerjee R (2014). Aging is associated with increased regulatory T-cell function. Aging Cell.

[204] Laumont CM, Nelson BH (2023). B cells in the tumor microenvironment: Multi-faceted organizers, regulators, and effectors of anti-tumor immunity. Cancer Cell.

[205] Carmi Y, Spitzer MH, Linde IL, Burt BM, Prestwood TR, Perlman N (2015). Allogeneic IgG combined with dendritic cell stimuli induce antitumour T-cell immunity. Nature.

[206] Chen J, Tan Y, Sun F, Hou L, Zhang C, Ge T (2020). Single-cell transcriptome and antigen-immunoglobin analysis reveals the diversity of B cells in non-small cell lung cancer. Genome Biol.

[207] Biswas S, Mandal G, Payne KK, Anadon CM, Gatenbee CD, Chaurio RA (2021). IgA transcytosis and antigen recognition govern ovarian cancer immunity. Nature.

[208] Fridman WH, Meylan M, Petitprez F, Sun CM, Italiano A, Sautès-Fridman C (2022). B cells and tertiary lymphoid structures as determinants of tumour immune contexture and clinical outcome. Nat Rev Clin Oncol.

[209] Sautès-Fridman C, Petitprez F, Calderaro J, Fridman WH (2019). Tertiary lymphoid structures in the era of cancer immunotherapy. Nat Rev Cancer.

[210] Souders CA, Bowers SL, Baudino TA (2009). Cardiac fibroblast: the renaissance cell. Circ Res.

[211] Kawamoto S, Uemura K, Hori N, Takayasu L, Konishi Y, Katoh K (2023). Bacterial induction of B cell senescence promotes age-related changes in the gut microbiota. Nat Cell Biol.

[212] Bulati M, Caruso C, Colonna-Romano G (2017). From lymphopoiesis to plasma cells differentiation, the age-related modifications of B cell compartment are influenced by "inflamm-ageing". Ageing Res Rev.

[213] Listì F, Candore G, Modica MA, Russo M, Di Lorenzo G, Esposito-Pellitteri M (2006). A study of serum immunoglobulin levels in elderly persons that provides new insights into B cell immunosenescence. Ann N Y Acad Sci.

[214] Henry CJ, Casás-Selves M, Kim J, Zaberezhnyy V, Aghili L, Daniel AE (2015). Aging-associated inflammation promotes selection for adaptive oncogenic events in B cell progenitors. J Clin Invest.

[215] Chen Y, McAndrews KM, Kalluri R (2021). Clinical and therapeutic relevance of cancer-associated fibroblasts. Nat Rev Clin Oncol.

[216] Mao X, Xu J, Wang W, Liang C, Hua J, Liu J (2021). Crosstalk between cancer-associated fibroblasts and immune cells in the tumor microenvironment: new findings and future perspectives. Mol Cancer.

[217] Ragunathan K, Upfold NLE, Oksenych V (2020). Interaction between Fibroblasts and Immune Cells Following DNA Damage Induced by Ionizing Radiation. Int J Mol Sci.

[218] Pazolli E, Alspach E, Milczarek A, Prior J, Piwnica-Worms D, Stewart SA (2012). Chromatin remodeling underlies the senescence-associated secretory phenotype of tumor stromal fibroblasts that supports cancer progression. Cancer Res.

[219] Yoshimoto S, Loo TM, Atarashi K, Kanda H, Sato S, Oyadomari S (2013). Obesity-induced gut microbial metabolite promotes liver cancer through senescence secretome. Nature.

[220] Yang G, Rosen DG, Zhang Z, Bast RC Jr, Mills GB, Colacino JA (2006). The chemokine growth-regulated oncogene 1 (Gro-1) links RAS signaling to the senescence of stromal fibroblasts and ovarian tumorigenesis. Proc Natl Acad Sci U S A.

[221] Golomb L, Sagiv A, Pateras IS, Maly A, Krizhanovsky V, Gorgoulis VG (2015). Age-associated inflammation connects RAS-induced senescence to stem cell dysfunction and epidermal malignancy. Cell Death Differ.

[222] Parrinello S, Coppe JP, Krtolica A, Campisi J (2005). Stromal-epithelial interactions in aging and cancer: senescent fibroblasts alter epithelial cell differentiation. J Cell Sci.

[223] Pazolli E, Luo X, Brehm S, Carbery K, Chung JJ, Prior JL (2009). Senescent stromal-derived osteopontin promotes preneoplastic cell growth. Cancer Res.

[224] Bavik C, Coleman I, Dean JP, Knudsen B, Plymate S, Nelson PS (2006). The gene expression program of prostate fibroblast senescence modulates neoplastic epithelial cell proliferation through paracrine mechanisms. Cancer Res.

[225] Krizhanovsky V, Yon M, Dickins RA, Hearn S, Simon J, Miething C (2008). Senescence of activated stellate cells limits liver fibrosis. Cell.

[226] Hassona Y, Cirillo N, Heesom K, Parkinson EK, Prime SS (2014). Senescent cancer-associated fibroblasts secrete active MMP-2 that promotes keratinocyte dis-cohesion and invasion. Br J Cancer.

[227] Wang T, Notta F, Navab R, Joseph J, Ibrahimov E, Xu J (2017). Senescent Carcinoma-Associated Fibroblasts Upregulate IL8 to Enhance Prometastatic Phenotypes. Mol Cancer Res.

[228] Ortiz-Montero P, Londoño-Vallejo A, Vernot JP (2017). Senescence-associated IL-6 and IL-8 cytokines induce a self- and cross-reinforced senescence/inflammatory milieu strengthening tumorigenic capabilities in the MCF-7 breast cancer cell line. Cell Commun Signal.

[229] Pili R, Guo Y, Chang J, Nakanishi H, Martin GR, Passaniti A (1994). Altered angiogenesis underlying age-dependent changes in tumor growth. J Natl Cancer Inst.

[230] Han L, Long Q, Li S, Xu Q, Zhang B, Dou X (2020). Senescent Stromal Cells Promote Cancer Resistance through SIRT1 Loss-Potentiated Overproduction of Small Extracellular Vesicles. Cancer Res.

[231] Takasugi M, Okada R, Takahashi A, Virya Chen D, Watanabe S, Hara E (2017). Small extracellular vesicles secreted from senescent cells promote cancer cell proliferation through EphA2. Nat Commun.

[232] Lehmann BD, Paine MS, Brooks AM, McCubrey JA, Renegar RH, Wang R (2008). Senescence-associated exosome release from human prostate cancer cells. Cancer Res.

[233] Jiang X, Wang J, Deng X, Xiong F, Zhang S, Gong Z (2020). The role of microenvironment in tumor angiogenesis. J Exp Clin Cancer Res.

[234] Olive KP, Jacobetz MA, Davidson CJ, Gopinathan A, McIntyre D, Honess D (2009). Inhibition of Hedgehog signaling enhances delivery of chemotherapy in a mouse model of pancreatic cancer. Science.

[235] Provenzano PP, Cuevas C, Chang AE, Goel VK, Von Hoff DD, Hingorani SR (2012). Enzymatic targeting of the stroma ablates physical barriers to treatment of pancreatic ductal adenocarcinoma. Cancer Cell.

[236] Assouline B, Kahn R, Hodali L, Condiotti R, Engel Y, Elyada E (2024). Senescent cancer-associated fibroblasts in pancreatic adenocarcinoma restrict CD8(+) T cell activation and limit responsiveness to immunotherapy in mice. Nat Commun.

[237] Fan G, Yu B, Tang L, Zhu R, Chen J, Zhu Y (2024). TSPAN8(+) myofibroblastic cancer-associated fibroblasts promote chemoresistance in patients with breast cancer. Sci Transl Med.

[238] Chen D, Liu P, Lin J, Zang L, Liu Y, Zhai S A Distinguished Roadmap of Fibroblast Senescence in Predicting Immunotherapy Response and Prognosis Across Human Cancers. Adv Sci (Weinh). 2024: e2406624.

[239] Liu L, Huang H, Cheng B, Xie H, Peng W, Cui H (2025). Revealing the role of cancer-associated fibroblast senescence in prognosis and immune landscape in pancreatic cancer. iScience.

[240] Wu Z, Uhl B, Gires O, Reichel CA (2023). A transcriptomic pan-cancer signature for survival prognostication and prediction of immunotherapy response based on endothelial senescence. J Biomed Sci.

[241] Grosse L, Wagner N, Emelyanov A, Molina C, Lacas-Gervais S, Wagner KD (2020). Defined p16(High) Senescent Cell Types Are Indispensable for Mouse Healthspan. Cell Metab.

[242] Hayashi T, Matsui-Hirai H, Miyazaki-Akita A, Fukatsu A, Funami J, Ding QF (2006). Endothelial cellular senescence is inhibited by nitric oxide: implications in atherosclerosis associated with menopause and diabetes. Proc Natl Acad Sci U S A.

[243] Matsushita H, Chang E, Glassford AJ, Cooke JP, Chiu CP, Tsao PS (2001). eNOS activity is reduced in senescent human endothelial cells: Preservation by hTERT immortalization. Circ Res.

[244] Zeng X, Wang TW, Yamaguchi K, Hatakeyama S, Yamazaki S, Shimizu E (2024). M2 macrophage-derived TGF-β induces age-associated loss of adipogenesis through progenitor cell senescence. Mol Metab.

[245] Benadjaoud MA, Soysouvanh F, Tarlet G, Paget V, Buard V, Santos de Andrade H (2022). Deciphering the Dynamic Molecular Program of Radiation-Induced Endothelial Senescence. Int J Radiat Oncol Biol Phys.

[246] Bent EH, Gilbert LA, Hemann MT (2016). A senescence secretory switch mediated by PI3K/AKT/mTOR activation controls chemoprotective endothelial secretory responses. Genes Dev.

[247] Ungvari Z, Podlutsky A, Sosnowska D, Tucsek Z, Toth P, Deak F (2013). Ionizing radiation promotes the acquisition of a senescence-associated secretory phenotype and impairs angiogenic capacity in cerebromicrovascular endothelial cells: role of increased DNA damage and decreased DNA repair capacity in microvascular radiosensitivity. J Gerontol A Biol Sci Med Sci.

[248] Mongiardi MP, Radice G, Piras M, Stagni V, Pacioni S, Re A (2019). Axitinib exposure triggers endothelial cells senescence through ROS accumulation and ATM activation. Oncogene.

[249] Wang D, Xiao F, Feng Z, Li M, Kong L, Huang L (2020). Sunitinib facilitates metastatic breast cancer spreading by inducing endothelial cell senescence. Breast Cancer Res.

[250] Usui S, Iso Y, Sasai M, Mizukami T, Mori H, Watanabe T (2014). Kisspeptin-10 induces endothelial cellular senescence and impaired endothelial cell growth. Clin Sci (Lond).

[251] Chang H, Rha SY, Jeung HC, Park KH, Kim TS, Kim YB (2013). Telomerase- and angiogenesis-related gene responses to irradiation in human umbilical vein endothelial cells. Int J Mol Med.

[252] Wieland E, Rodriguez-Vita J, Liebler SS, Mogler C, Moll I, Herberich SE (2017). Endothelial Notch1 Activity Facilitates Metastasis. Cancer Cell.

[253] Castellani G, Buccarelli M, D'Alessandris QG, Ilari R, Cappannini A, Pedini F (2024). Extracellular vesicles produced by irradiated endothelial or Glioblastoma stem cells promote tumor growth and vascularization modulating tumor microenvironment. Cancer Cell Int.

[254] Zhu Z, Cao Q, Chen J, Sun Y, Liu F, Li J (2025). Expression pattern of cancer-associated cellular senescence genes in clear cell renal cell carcinoma distinguishes tumor subclasses with clinical implications. Sci Rep.

[255] Coppé JP, Rodier F, Patil CK, Freund A, Desprez PY, Campisi J (2011). Tumor suppressor and aging biomarker p16(INK4a) induces cellular senescence without the associated inflammatory secretory phenotype. J Biol Chem.

[256] Wang B, Han J, Elisseeff JH, Demaria M (2024). The senescence-associated secretory phenotype and its physiological and pathological implications. Nat Rev Mol Cell Biol.

[257] Eggert T, Wolter K, Ji JL, Ma C, Yevsa T, Klotz S (2016). Distinct Functions of Senescence-Associated Immune Responses in Liver Tumor Surveillance and Tumor Progression. Cancer Cell.

[258] Cheng Y, Li H, Deng Y, Tai Y, Zeng K, Zhang Y (2018). Cancer-associated fibroblasts induce PDL1+ neutrophils through the IL6-STAT3 pathway that foster immune suppression in hepatocellular carcinoma. Cell Death Dis.

[259] Cheng JT, Deng YN, Yi HM, Wang GY, Fu BS, Chen WJ (2016). Hepatic carcinoma-associated fibroblasts induce IDO-producing regulatory dendritic cells through IL-6-mediated STAT3 activation. Oncogenesis.

[260] Deng Y, Cheng J, Fu B, Liu W, Chen G, Zhang Q (2017). Hepatic carcinoma-associated fibroblasts enhance immune suppression by facilitating the generation of myeloid-derived suppressor cells. Oncogene.

[261] Mace TA, Ameen Z, Collins A, Wojcik S, Mair M, Young GS (2013). Pancreatic cancer-associated stellate cells promote differentiation of myeloid-derived suppressor cells in a STAT3-dependent manner. Cancer Res.

[262] Zhao Q, Huang L, Qin G, Qiao Y, Ren F, Shen C (2021). Cancer-associated fibroblasts induce monocytic myeloid-derived suppressor cell generation via IL-6/exosomal miR-21-activated STAT3 signaling to promote cisplatin resistance in esophageal squamous cell carcinoma. Cancer Lett.

[263] Ye J, Baer JM, Faget DV, Morikis VA, Ren Q, Melam A (2024). Senescent CAFs Mediate Immunosuppression and Drive Breast Cancer Progression. Cancer Discov.

[264] Kato T, Noma K, Ohara T, Kashima H, Katsura Y, Sato H (2018). Cancer-Associated Fibroblasts Affect Intratumoral CD8(+) and FoxP3(+) T Cells Via IL6 in the Tumor Microenvironment. Clin Cancer Res.

[265] Song M, He J, Pan QZ, Yang J, Zhao J, Zhang YJ (2021). Cancer-Associated Fibroblast-Mediated Cellular Crosstalk Supports Hepatocellular Carcinoma Progression. Hepatology.

[266] Viel S, Marçais A, Guimaraes FS, Loftus R, Rabilloud J, Grau M (2016). TGF-β inhibits the activation and functions of NK cells by repressing the mTOR pathway. Sci Signal.

[267] Chen W, Jin W, Hardegen N, Lei KJ, Li L, Marinos N (2003). Conversion of peripheral CD4+CD25- naive T cells to CD4+CD25+ regulatory T cells by TGF-beta induction of transcription factor Foxp3. J Exp Med.

[268] Gutcher I, Donkor MK, Ma Q, Rudensky AY, Flavell RA, Li MO (2011). Autocrine transforming growth factor-β1 promotes in vivo Th17 cell differentiation. Immunity.

[269] Thomas DA, Massagué J (2005). TGF-beta directly targets cytotoxic T cell functions during tumor evasion of immune surveillance. Cancer Cell.

[270] Morad G, Helmink BA, Sharma P, Wargo JA (2021). Hallmarks of response, resistance, and toxicity to immune checkpoint blockade. Cell.

[271] Colucci M, Zumerle S, Bressan S, Gianfanti F, Troiani M, Valdata A (2024). Retinoic acid receptor activation reprograms senescence response and enhances anti-tumor activity of natural killer cells. Cancer Cell.

[272] Chibaya L, DeMarco KD, Lusi CF, Kane GI, Brassil ML, Parikh CN (2024). Nanoparticle delivery of innate immune agonists combined with senescence-inducing agents promotes T cell control of pancreatic cancer. Sci Transl Med.

[273] Wang Z, Chen Y, Fang H, Xiao K, Wu Z, Xie X (2024). Reprogramming cellular senescence in the tumor microenvironment augments cancer immunotherapy through multifunctional nanocrystals. Sci Adv.

[274] Liu Y, Pagacz J, Wolfgeher DJ, Bromerg KD, Gorman JV, Kron SJ (2023). Senescent cancer cell vaccines induce cytotoxic T cell responses targeting primary tumors and disseminated tumor cells. J Immunother Cancer.

[275] Mongiardi MP, Pellegrini M, Pallini R, Levi A, Falchetti ML (2021). Cancer Response to Therapy-Induced Senescence: A Matter of Dose and Timing. Cancers (Basel).

[276] Wang C, Vegna S, Jin H, Benedict B, Lieftink C, Ramirez C (2019). Inducing and exploiting vulnerabilities for the treatment of liver cancer. Nature.

[277] Zhang Y, Xiao B, Yuan S, Ding L, Pan Y, Jiang Y (2024). Tryptanthrin targets GSTP1 to induce senescence and increases the susceptibility to apoptosis by senolytics in liver cancer cells. Redox Biol.

[278] Toso A, Revandkar A, Di Mitri D, Guccini I, Proietti M, Sarti M (2014). Enhancing chemotherapy efficacy in Pten-deficient prostate tumors by activating the senescence-associated antitumor immunity. Cell Rep.

[279] Georgilis A, Klotz S, Hanley CJ, Herranz N, Weirich B, Morancho B (2018). PTBP1-Mediated Alternative Splicing Regulates the Inflammatory Secretome and the Pro-tumorigenic Effects of Senescent Cells. Cancer Cell.

[280] Nacarelli T, Lau L, Fukumoto T, Zundell J, Fatkhutdinov N, Wu S (2019). NAD(+) metabolism governs the proinflammatory senescence-associated secretome. Nat Cell Biol.

[281] Li F, Huangyang P, Burrows M, Guo K, Riscal R, Godfrey J (2020). FBP1 loss disrupts liver metabolism and promotes tumorigenesis through a hepatic stellate cell senescence secretome. Nat Cell Biol.

[282] Pernicova I, Korbonits M (2014). Metformin-mode of action and clinical implications for diabetes and cancer. Nat Rev Endocrinol.

[283] Yang J, Liu HC, Zhang JQ, Zou JY, Zhang X, Chen WM (2023). The effect of metformin on senescence of T lymphocytes. Immun Ageing.

[284] Kulkarni AS, Gubbi S, Barzilai N (2020). Benefits of Metformin in Attenuating the Hallmarks of Aging. Cell Metab.

[285] Barzilai N, Crandall JP, Kritchevsky SB, Espeland MA (2016). Metformin as a Tool to Target Aging. Cell Metab.

[286] Herranz N, Gallage S, Mellone M, Wuestefeld T, Klotz S, Hanley CJ (2015). mTOR regulates MAPKAPK2 translation to control the senescence-associated secretory phenotype. Nat Cell Biol.

[287] Herranz N, Gallage S, Mellone M, Wuestefeld T, Klotz S, Hanley CJ (2015). Erratum: mTOR regulates MAPKAPK2 translation to control the senescence-associated secretory phenotype. Nat Cell Biol.

[288] Laberge RM, Sun Y, Orjalo AV, Patil CK, Freund A, Zhou L (2015). MTOR regulates the pro-tumorigenic senescence-associated secretory phenotype by promoting IL1A translation. Nat Cell Biol.

[289] Laberge RM, Sun Y, Orjalo AV, Patil CK, Freund A, Zhou L (2021). Author Correction: MTOR regulates the pro-tumorigenic senescence-associated secretory phenotype by promoting IL1A translation. Nat Cell Biol.

[290] Mannick JB, Morris M, Hockey HP, Roma G, Beibel M, Kulmatycki K (2018). TORC1 inhibition enhances immune function and reduces infections in the elderly. Sci Transl Med.

[291] Bitto A, Ito TK, Pineda VV, LeTexier NJ, Huang HZ, Sutlief E (2016). Transient rapamycin treatment can increase lifespan and healthspan in middle-aged mice. Elife.

[292] Davis SL, Ionkina AA, Bagby SM, Orth JD, Gittleman B, Marcus JM (2020). Preclinical and Dose-Finding Phase I Trial Results of Combined Treatment with a TORC1/2 Inhibitor (TAK-228) and Aurora A Kinase Inhibitor (Alisertib) in Solid Tumors. Clin Cancer Res.

[293] Cleary JM, Lima CM, Hurwitz HI, Montero AJ, Franklin C, Yang J (2014). A phase I clinical trial of navitoclax, a targeted high-affinity Bcl-2 family inhibitor, in combination with gemcitabine in patients with solid tumors. Invest New Drugs.

[294] Puglisi M, Molife LR, de Jonge MJ, Khan KH, Doorn LV, Forster MD (2021). A Phase I study of the safety, pharmacokinetics and efficacy of navitoclax plus docetaxel in patients with advanced solid tumors. Future Oncol.

[295] de Vos S, Leonard JP, Friedberg JW, Zain J, Dunleavy K, Humerickhouse R (2021). Safety and efficacy of navitoclax, a BCL-2 and BCL-X(L) inhibitor, in patients with relapsed or refractory lymphoid malignancies: results from a phase 2a study. Leuk Lymphoma.

[296] Kipps TJ, Eradat H, Grosicki S, Catalano J, Cosolo W, Dyagil IS (2015). A phase 2 study of the BH3 mimetic BCL2 inhibitor navitoclax (ABT-263) with or without rituximab, in previously untreated B-cell chronic lymphocytic leukemia. Leuk Lymphoma.

[297] Chaib S, Tchkonia T, Kirkland JL (2022). Cellular senescence and senolytics: the path to the clinic. Nat Med.

[298] Carpenter VJ, Saleh T, Gewirtz DA (2021). Senolytics for Cancer Therapy: Is All That Glitters Really Gold?. Cancers (Basel).

[299] Wang Y, Che L, Chen X, He Z, Song D, Yuan Y (2023). Repurpose dasatinib and quercetin: Targeting senescent cells ameliorates postmenopausal osteoporosis and rejuvenates bone regeneration. Bioact Mater.

[300] Novais EJ, Tran VA, Johnston SN, Darris KR, Roupas AJ, Sessions GA (2021). Long-term treatment with senolytic drugs Dasatinib and Quercetin ameliorates age-dependent intervertebral disc degeneration in mice. Nat Commun.

[301] Hickson LJ, Langhi Prata LGP, Bobart SA, Evans TK, Giorgadze N, Hashmi SK (2019). Senolytics decrease senescent cells in humans: Preliminary report from a clinical trial of Dasatinib plus Quercetin in individuals with diabetic kidney disease. EBioMedicine.

[302] Pastor-Fernández A, Bertos AR, Sierra-Ramírez A, Del Moral-Salmoral J, Merino J, de Ávila AI (2023). Treatment with the senolytics dasatinib/quercetin reduces SARS-CoV-2-related mortality in mice. Aging Cell.

[303] Bientinesi E, Ristori S, Lulli M, Monti D (2024). Quercetin induces senolysis of doxorubicin-induced senescent fibroblasts by reducing autophagy, preventing their pro-tumour effect on osteosarcoma cells. Mech Ageing Dev.

[304] Wang L, Xiong B, Lu W, Cheng Y, Zhu J, Ai G (2024). Senolytic drugs dasatinib and quercetin combined with Carboplatin or Olaparib reduced the peritoneal and adipose tissue metastasis of ovarian cancer. Biomed Pharmacother.

[305] Khan S, Cao L, Wiegand J, Zhang P, Zajac-Kaye M, Kaye FJ (2024). PROTAC-Mediated Dual Degradation of BCL-xL and BCL-2 Is a Highly Effective Therapeutic Strategy in Small-Cell Lung Cancer. Cells.

[306] Demaria M, O'Leary MN, Chang J, Shao L, Liu S, Alimirah F (2017). Cellular Senescence Promotes Adverse Effects of Chemotherapy and Cancer Relapse. Cancer Discov.

[307] Vlahovic G, Karantza V, Wang D, Cosgrove D, Rudersdorf N, Yang J (2014). A phase I safety and pharmacokinetic study of ABT-263 in combination with carboplatin/paclitaxel in the treatment of patients with solid tumors. Invest New Drugs.

[308] Szymczak J, Cielecka-Piontek J (2023). Fisetin-In Search of Better Bioavailability-From Macro to Nano Modifications: A Review. Int J Mol Sci.

[309] Chen YX, Wang CJ, Xiao DS, He BM, Li M, Yi XP (2021). eIF3a R803K mutation mediates chemotherapy resistance by inducing cellular senescence in small cell lung cancer. Pharmacol Res.

[310] Jochems F, Baltira C, MacDonald JA, Daniels V, Mathur A, de Gooijer MC (2024). Senolysis by ABT-263 is associated with inherent apoptotic dependence of cancer cells derived from the non-senescent state. Cell Death Differ.

[311] Jochems F, Thijssen B, De Conti G, Jansen R, Pogacar Z, Groot K (2021). The Cancer SENESCopedia: A delineation of cancer cell senescence. Cell Rep.

[312] Alcon C, Kovatcheva M, Morales-Sánchez P, López-Polo V, Torres T, Puig S (2024). HRK downregulation and augmented BCL-xL binding to BAK confer apoptotic protection to therapy-induced senescent melanoma cells. Cell Death Differ.

[313] Kovacovicova K, Skolnaja M, Heinmaa M, Mistrik M, Pata P, Pata I (2018). Senolytic Cocktail Dasatinib+Quercetin (D+Q) Does Not Enhance the Efficacy of Senescence-Inducing Chemotherapy in Liver Cancer. Front Oncol.

[314] Ji P, Wang C, Liu Y, Guo X, Liang Y, Wei J (2024). Targeted Clearance of Senescent Cells Via Engineered Extracellular Vesicles Reprograms Tumor Immunosuppressive Microenvironment. Adv Healthc Mater.

[315] Zhang H, Xu X, Shou X, Liao W, Jin C, Chen C (2024). Senolytic Therapy Enabled by Senescent Cell-Sensitive Biomimetic Melanin Nano-Senolytics. Adv Healthc Mater.

[316] Parshad B, Baker AG, Ahmed I, Estepa-Fernández A, Muñoz-Espín D, Fruk L Improved Therapeutic Efficiency of Senescent Cell-specific, Galactose-Functionalized Micelle Nanocarriers. Small. 2024: e2405732.

[317] Chang M, Dong Y, Xu H, Cruickshank-Taylor AB, Kozora JS, Behpour B (2024). Senolysis Enabled by Senescent Cell-Sensitive Bioorthogonal Tetrazine Ligation. Angew Chem Int Ed Engl.

[318] Chang M, Gao F, Gnawali G, Xu H, Dong Y, Meng X (2024). Selective Elimination of Senescent Cancer Cells by Galacto-Modified PROTACs. J Med Chem.

[319] Peng Y, Liu D, Huang D, Inuzuka H, Liu J (2024). PROTAC as a novel anti-cancer strategy by targeting aging-related signaling. Semin Cancer Biol.

[320] McHugh D, Durán I, Gil J (2025). Senescence as a therapeutic target in cancer and age-related diseases. Nat Rev Drug Discov.

[321] Yang Y, Jn-Simon N, He Y, Sun C, Zhang P, Hu W (2025). A BCL-xL/BCL-2 PROTAC effectively clears senescent cells in the liver and reduces MASH-driven hepatocellular carcinoma in mice. Nat Aging.

[322] Amor C, Fernández-Maestre I, Chowdhury S, Ho YJ, Nadella S, Graham C (2024). Prophylactic and long-lasting efficacy of senolytic CAR T cells against age-related metabolic dysfunction. Nat Aging.

[323] Ming X, Yang Z, Huang Y, Wang Z, Zhang Q, Lu C (2024). A chimeric peptide promotes immune surveillance of senescent cells in injury, fibrosis, tumorigenesis and aging. Nat Aging.

[324] Yang D, Sun B, Li S, Wei W, Liu X, Cui X (2023). NKG2D-CAR T cells eliminate senescent cells in aged mice and nonhuman primates. Sci Transl Med.

[325] Stirrups R (2018). CAR T-cells for relapsed B-cell ALL in children and young adults. Lancet Oncol.

[326] Zhu X, Prasad S, Gaedicke S, Hettich M, Firat E, Niedermann G (2015). Patient-derived glioblastoma stem cells are killed by CD133-specific CAR T cells but induce the T cell aging marker CD57. Oncotarget.

[327] Roselle C, Horikawa I, Chen L, Kelly AR, Gonzales D, Da T (2024). Enhancing chimeric antigen receptor T cell therapy by modulating the p53 signaling network with Δ133p53α. Proc Natl Acad Sci U S A.

[328] Yao GD, Yang J, Li XX, Song XY, Hayashi T, Tashiro SI (2017). Blocking the utilization of glucose induces the switch from senescence to apoptosis in pseudolaric acid B-treated human lung cancer cells in vitro. Acta Pharmacol Sin.

[329] He Y, Liu Y, Zheng M, Zou Y, Huang M, Wang L (2025). Targeting ATAD3A Phosphorylation Mediated by TBK1 Ameliorates Senescence-Associated Pathologies. Adv Sci (Weinh).

[330] Jochems F, Baltira C, MacDonald JA, Daniels V, Mathur A, de Gooijer MC (2025). Senolysis by ABT-263 is associated with inherent apoptotic dependence of cancer cells derived from the non-senescent state. Cell Death Differ.

[331] MacDonald JA, Bradshaw GA, Jochems F, Bernards R, Letai A (2025). Apoptotic priming in senescence predicts specific senolysis by quantitative analysis of mitochondrial dependencies. Cell Death Differ.

[332] Baldwin JG, Heuser-Loy C, Saha T, Schelker RC, Slavkovic-Lukic D, Strieder N (2024). Intercellular nanotube-mediated mitochondrial transfer enhances T cell metabolic fitness and antitumor efficacy. Cell.

[333] Yoshida S, Nakagami H, Hayashi H, Ikeda Y, Sun J, Tenma A (2020). The CD153 vaccine is a senotherapeutic option for preventing the accumulation of senescent T cells in mice. Nat Commun.

[334] Suda M, Shimizu I, Katsuumi G, Yoshida Y, Hayashi Y, Ikegami R (2021). Senolytic vaccination improves normal and pathological age-related phenotypes and increases lifespan in progeroid mice. Nat Aging.

[335] Zhou Z, Yao J, Wu D, Huang X, Wang Y, Li X (2024). Type 2 cytokine signaling in macrophages protects from cellular senescence and organismal aging. Immunity.

[336] Wang T, Liu W, Shen Q, Tao R, Li C, Shen Q (2023). Combination of PARP inhibitor and CDK4/6 inhibitor modulates cGAS/STING-dependent therapy-induced senescence and provides "one-two punch" opportunity with anti-PD-L1 therapy in colorectal cancer. Cancer Sci.

[337] Zhang J, Zhang S, Cheng C, Zhu C, Wang T, Tang L (2025). Targeting senescence with radioactive (223)Ra/Ba SAzymes enables senolytics-unlocked One-Two punch strategy to boost anti-tumor immunotherapy. Biomaterials.

[338] Wang H, Yuan S, Zheng Q, Zhang S, Zhang Q, Ji S (2024). Dual Inhibition of CDK4/6 and XPO1 Induces Senescence With Acquired Vulnerability to CRBN-Based PROTAC Drugs. Gastroenterology.

[339] Bi Y, Qiao X, Cai Z, Zhao H, Ye R, Liu Q (2025). Exosomal miR-302b rejuvenates aging mice by reversing the proliferative arrest of senescent cells. Cell Metab.

[340] Gu L, Zhu Y, Nandi SP, Lee M, Watari K, Bareng B (2025). FBP1 controls liver cancer evolution from senescent MASH hepatocytes. Nature.

[341] Henson SM, Snelgrove R, Hussell T, Wells DJ, Aspinall R (2005). An IL-7 fusion protein that shows increased thymopoietic ability. J Immunol.

[342] Al-Chami E, Tormo A, Pasquin S, Kanjarawi R, Ziouani S, Rafei M (2016). Interleukin-21 administration to aged mice rejuvenates their peripheral T-cell pool by triggering de novo thymopoiesis. Aging Cell.

[343] Tormo A, Khodayarian F, Cui Y, Al-Chami E, Kanjarawi R, Noé B (2017). Interleukin-21 promotes thymopoiesis recovery following hematopoietic stem cell transplantation. J Hematol Oncol.

[344] Sun L, Guo J, Brown R, Amagai T, Zhao Y, Su DM (2010). Declining expression of a single epithelial cell-autonomous gene accelerates age-related thymic involution. Aging Cell.

[345] Li J, Wachsmuth LP, Xiao S, Condie BG, Manley NR (2023). Foxn1 overexpression promotes thymic epithelial progenitor cell proliferation and mTEC maintenance, but does not prevent thymic involution. Development.

[346] Tuckett AZ, Thornton RH, O'Reilly RJ, van den Brink MRM, Zakrzewski JL (2017). Intrathymic injection of hematopoietic progenitor cells establishes functional T cell development in a mouse model of severe combined immunodeficiency. J Hematol Oncol.

[347] Politikos I, Boussiotis VA (2014). The role of the thymus in T-cell immune reconstitution after umbilical cord blood transplantation. Blood.

[348] Nishimura T, Kaneko S, Kawana-Tachikawa A, Tajima Y, Goto H, Zhu D (2013). Generation of rejuvenated antigen-specific T cells by reprogramming to pluripotency and redifferentiation. Cell Stem Cell.

[349] Licastro F, Candore G, Lio D, Porcellini E, Colonna-Romano G, Franceschi C (2005). Innate immunity and inflammation in ageing: a key for understanding age-related diseases. Immun Ageing.

[350] Jaillon S, Ponzetta A, Di Mitri D, Santoni A, Bonecchi R, Mantovani A (2020). Neutrophil diversity and plasticity in tumour progression and therapy. Nat Rev Cancer.

[351] Sun R, Luo J, Li D, Shu Y, Luo C, Wang SS (2014). Neutrophils with protumor potential could efficiently suppress tumor growth after cytokine priming and in presence of normal NK cells. Oncotarget.

[352] Wang TT, Zhao YL, Peng LS, Chen N, Chen W, Lv YP (2017). Tumour-activated neutrophils in gastric cancer foster immune suppression and disease progression through GM-CSF-PD-L1 pathway. Gut.

[353] Suryadevara V, Hudgins AD, Rajesh A, Pappalardo A, Karpova A, Dey AK (2024). SenNet recommendations for detecting senescent cells in different tissues. Nat Rev Mol Cell Biol.

[354] Ogrodnik M, Carlos Acosta J, Adams PD, d'Adda di Fagagna F, Baker DJ, Bishop CL (2024). Guidelines for minimal information on cellular senescence experimentation in vivo. Cell.

[355] El-Sadoni M, Shboul SA, Alhesa A, Shahin NA, Alsharaiah E, Ismail MA (2023). A three-marker signature identifies senescence in human breast cancer exposed to neoadjuvant chemotherapy. Cancer Chemother Pharmacol.

[356] Saleh T, Alhesa A, El-Sadoni M, Abu Shahin N, Alsharaiah E, Al Shboul S (2022). The Expression of the Senescence-Associated Biomarker Lamin B1 in Human Breast Cancer. Diagnostics (Basel).

[357] Kim KM, Noh JH, Bodogai M, Martindale JL, Yang X, Indig FE (2017). Identification of senescent cell surface targetable protein DPP4. Genes Dev.

[358] Smer-Barreto V, Quintanilla A, Elliott RJR, Dawson JC, Sun J, Campa VM (2023). Discovery of senolytics using machine learning. Nat Commun.

[359] Duran I, Pombo J, Sun B, Gallage S, Kudo H, McHugh D (2024). Detection of senescence using machine learning algorithms based on nuclear features. Nat Commun.

[360] Zhao H, Liu Z, Chen H, Han M, Zhang M, Liu K (2024). Identifying specific functional roles for senescence across cell types. Cell.

